# A Hybrid Method to Solve the Multi-UAV Dynamic Task Assignment Problem

**DOI:** 10.3390/s25082502

**Published:** 2025-04-16

**Authors:** Shahad Alqefari, Mohamed El Bachir Menai

**Affiliations:** 1 Department of Computer Science, College of Computer and Information Science, King Saud University, Riyadh 11451, Saudi Arabia; menai@ksu.edu.sa; 2 Department of Computer Science, College of Computer and Information Science, Imam Mohammed Ibn Saud Islamic University, Riyadh 11564, Saudi Arabia

**Keywords:** multi-UAV systems, dynamic task allocation, hybrid algorithm, UAV clustering techniques, real-time UAV coordination

## Abstract

In the rapidly evolving field of aerial robotics, the coordinated management of multiple unmanned aerial vehicle (multi-UAV) systems to address complex and dynamic environments is increasingly critical. Multi-UAV systems promise enhanced efficiency and effectiveness in various applications, from disaster response to infrastructure inspection, by leveraging the collective capabilities of UAV fleets. However, the dynamic nature of such environments presents significant challenges in task allocation and real-time adaptability. This paper introduces a novel hybrid algorithm designed to optimize multi-UAV task assignments in dynamic environments. State-of-the-art solutions in this domain have exhibited limitations, particularly in rapidly responding to dynamic changes and effectively scaling to large-scale environments. The proposed solution bridges these gaps by combining clustering to group and assign tasks in an initial offline phase with a dynamic partial reassignment process that locally updates assignments in response to real-time changes, all within a centralized–distributed communication topology. The simulation results validate the superiority of the proposed solution and demonstrate its improvements in efficiency and responsiveness over existing solutions. Additionally, the results highlight the scalability of the solution in handling large-scale problems and demonstrate its ability to efficiently manage a growing number of UAVs and tasks. It also demonstrated robust adaptability and enhanced mission effectiveness across a wide range of dynamic events and different scale scenarios.

## 1. Introduction

Deploying a group of robots in a cooperative manner to accomplish a set of tasks represents a significant research area in the robotics domain [1,2,3]. These systems, known as multirobot systems (MRSs), leverage the cooperative behavior of robots to complete complex tasks that are time-consuming and exhausting for a single robot [2,4]. A key challenge in MRSs is the multirobot task allocation (MRTA) problem, which seeks to optimally assign a set of robots a series of tasks such that predefined objectives and overall performance are maximized, while respecting a set of constraints [5].

The advancement of UAV technology has become one of the most transformative developments in autonomous intelligent systems in the 21st century, drawing considerable attention from the research community [6]. UAVs have demonstrated significant value across a wide range of real-world applications, including reconnaissance missions [7], disaster response and recovery [8], communication support [9], surveillance operations [10], and autonomous exploration tasks [11]. While single-UAV systems face limitations in terms of coverage, adaptability, and fault tolerance, multi-UAV systems—where multiple UAVs collaborate to accomplish complex missions—offer several key advantages. These include distributed workload management, the integration of diverse UAV capabilities, enhanced resilience to single-point failures, and improved scalability for large-scale problem instances [12,13]. Such benefits make multi-UAV systems particularly well-suited for dynamic, mission-critical environments, and highlight their growing significance in both academic research and practical deployment.

The multi-UAV task assignment problem, a specific instance of the MRTA problem, involves a set of UAVs being tasked with performing a series of assignments where the objective is to find an optimal match between the UAVs and the tasks, while satisfying predefined constraints [14,15,16,17]. This is a combinatorial optimization problem known to be NP-hard [7,18], with the complexity and computational requirements increasing significantly as the number of tasks and UAVs grows [5,18,19,20].

Many of the existing studies [21,22,23,24,25] focused on the static version of the multi-UAV task assignment problem, where all tasks are predetermined and the environment remains unchanged [26]. However, in reality, environments are dynamic and unpredictable due to the occurrence of various dynamic events [7], such as the arrival of new tasks or UAV failures. These events require rapid and flexible responses and make the dynamic version of this problem particularly challenging [27]. Existing solutions to the dynamic multi-UAV task assignment problem often struggle to adapt quickly and efficiently to such changes, highlighting a significant gap in current research.

To address these challenges, this paper proposes a novel hybrid algorithm that combines clustering algorithms with partial reassignment algorithms, enhanced by a communication topology that integrates both centralized and distributed structures. This solution aims to maximize responsiveness, increase scalability, and improve robustness. The primary contributions of this paper are threefold:

1. Clustering for Large-Scale Environments: We use a clustering approach that segments the operational environment into smaller, manageable areas and distributes UAV resources in a balanced manner based on the task density within each cluster. Unlike existing global assignment approaches that allocate UAVs without considering localized workload variations, our method ensures that task allocation is performed locally within each cluster in a decentralized manner. This significantly improves scalability by reducing the computational overhead, and enhances responsiveness by enabling the UAVs to focus on nearby tasks, reducing travel time and improving real-time adaptability.
2. Partial Reassignment Strategy for Fast and Adaptive Task Handling: The proposed solution employs a partial reassignment strategy that selectively engages only the nearest subset of UAVs when a dynamic event occurs. These UAVs are released from a portion of the farthest assigned tasks, prioritizing proximity to the new task to minimize travel delays and maximize reassignment efficiency. Unlike traditional reassignment approaches that either reset all task assignments (e.g., the Consensus-Based Bundle Algorithm (CBBA) full reset) or rely on static heuristics, our method ensures that reallocation is both targeted and efficient, reducing unnecessary disruptions. This enhances robustness, allowing UAVs to adapt quickly to dynamic changes, while maintaining high operational continuity and minimizing interruptions to ongoing tasks.
3. Intelligent Idle UAV Management for Efficient Resource Utilization: The proposed solution introduces a centralized idle UAV management mechanism to ensure efficient resource utilization and maximize task coverage. When a UAV becomes idle, the central controller first checks for any unassigned tasks and directs the UAV to handle them immediately. If no unassigned tasks exist, the controller reallocates the idle UAV to assist the most heavily loaded UAV within its cluster, ensuring workload balancing. If the cluster is fully covered, the idle UAV is relocated to the nearest uncovered cluster to minimize travel time and enhance mission efficiency. Unlike existing methods that either leave idle UAVs underutilized or lack a structured relocation strategy, our approach proactively redistributes UAVs based on real-time task demand. This optimizes resource allocation, accelerates task completion, and enhances overall system scalability and responsiveness in dynamic environments.
4. Hybrid Centralized-Distributed Communication for Efficient Coordination: The proposed communication topology optimally balances centralized coordination with distributed execution, significantly reducing communication overhead. Within each cluster, UAVs exchange data using a distributed structure, leveraging decentralized task assignment algorithms such as CBBA, Performance Impact Algorithm (PI), or similar approaches to minimize excessive inter-UAV communication. At the same time, the central controller manages inter-cluster coordination, ensuring seamless high-level decision-making, while restricting unnecessary global data exchange. This dual-layer approach accelerates responses to ongoing tasks and dynamic events, avoiding the bottlenecks associated with fully centralized methods, while maintaining better organization and efficiency than purely decentralized systems. As a result, the proposed communication strategy enhances responsiveness, reduces delays, and improves overall mission execution in complex operational environments.

The remainder of this paper is organized as follows: Section 2 provides a detailed overview of the state-of-the-art solutions and their limitations. Section 3 describes the problem definition and the mathematical formulation of the dynamic multi-UAV task assignment problem. Section 4 details the proposed hybrid solution, emphasizing its algorithm design and operational advantages. Section 5 presents the simulation setup and results, and demonstrates the performance of the proposed solution. Finally, Section 6 concludes the paper with a summary of our findings and potential directions for future research.

## 2. Literature Review

The literature recognizes three primary solution approaches to the dynamic task assignment problem—market-based algorithms, optimization-based algorithms, and cluster algorithms—with extensive research applied to multi-UAV systems.

### 2.1. Market-Based Algorithms

Market-based algorithms are favored for their compatibility with the distributed structures of multi-UAV systems. This approach is inspired by the market trading–auction concept, which provides a communication mechanism between agents to coordinate information exchange [28,29].

Common methods based on market mechanisms include auction algorithms and contract net protocols. In practice, auction algorithms [30] assign UAVs to tasks by simulating the auction process. CBBA, proposed by Choi [31], is considered a multiagent task allocation algorithm. Extensions to CBBA aimed to enhance efficiency; one notable improvement is the insertion of tasks during UAV idle periods through an auction mechanism [32]. However, the new task is handled based on the availability of free idle time slots after checking all UAVs, which is computationally extensive. Another adaptation, CBBA with local replanning (CBBA-LR) [33], allows UAVs with spare resources to dynamically replan tasks. Other extended strategies for handling new tasks include no reset [31], full reset [34], single reset [35], heuristic full reset [35], and partial replanning (PR) [35]. The no reset strategy quickly adds new tasks without altering existing assignments but may result in suboptimal task allocations or degraded performance under dynamic conditions. The full reset strategy overhauls task assignments using the CBBA for a better quality at the expense of the response time. The single reset strategy modifies one task when a UAV’s capacity is full, while heuristic full reset uses a decision rule to choose optimal resets based on potential gains. The PR strategy balances quality and speed by partially resetting tasks before applying the CBBA, with variations for equal or strategic task resets based solely on initial assignments. Wu et al. [36] extended the CBBA using a distributed genetic algorithm to optimize operation times and communication in disaster relief scenarios. However, many of these methods have not been evaluated in large-scale environments and lack demonstrated effectiveness in handling complex, real-time task dynamics.

The performance impact (PI) algorithm [37], inspired by the CBBA, and its extension, the PI-MaxAss algorithm [38], focus on reallocating tasks among UAVs to create slots for unallocated tasks, and aim to enhance both the execution speed and task allocation without restarting the process. Other research [18] enhanced the baseline PI algorithm by integrating dynamic rescheduling capabilities and improving exploration properties to effectively avoid local minima in real-time scenarios. However, in these studies, the entire UAV swarm was involved in every new task allocation, without a clear communication structure. The Distributed Allocation with Time Windows (DATW) algorithm proposed in [39] integrates time window constraints within a PI-based framework, and emphasizes efficient and conflict-free task reallocation through an iterative approach. However, its comprehensive communication requirements can cause delays in urgent scenarios. The study in [40] developed a compromised PI Algorithm for UAV task allocation in search and rescue operations. It addresses new tasks dynamically but lacks detailed explanations of UAV negotiations for achieving consensus on task reassignments.

Studies have enhanced the contract net protocol (CNP) [41], including the two-stage distributed task assignment algorithm (TS-DTA) [42], which is designed to efficiently address changes such as UAV damage and target shifts by facilitating rapid task reassignment, while reducing communication burdens among UAVs. Additionally, a framework utilizing the CNP and Non-dominated Sorting Genetic Algorithm (NSGA-II) [43] proposed rapid task reassignment with reduced communication loads. However, in these studies, as the complexity of the problem increased, the volume of UAV communication increased, potentially compromising the system’s reliability. This paper [44] introduces an algorithm that adapts the CNP to dynamically manage UAV tasks and environmental changes using a hierarchical strategy and rapid-exploration random tree for path replanning. However, extensive UAV participation leads to significant negotiation delays, and the system does not effectively utilize idle or failed UAVs.

### 2.2. Intelligent Optimization Algorithms

Intelligent optimization algorithms, based on natural phenomena or social behavior, aim to identify the best solution from feasible options, subject to constraints, and focus on evolutionary and swarm intelligence algorithms such as genetic algorithms [45] and particle swarm optimization (PSO) [46].

The hybrid architecture proposed in [47] combines an improved discrete PSO with a market auction mechanism to address multi-UAV dynamic task allocation. Other research [48] introduced a novel randomized particle swarm optimizer (RPSO) that employs Gaussian white noise to enhance exploration and escape local optima more effectively. This paper [49] proposes the Improved Elite Parallel Particle Swarm Optimization (IEPPSO) algorithm, which incorporates time window constraints into the established task allocation model and integrates a probability-based constraint verification strategy and an experience pool updating mechanism.

The study in [50] proposed a multidiscrete wolf pack algorithm to enhance traditional approaches to dynamic multi-UAV task assignment. Peng et al. [51] introduced a binary wolf pack algorithm for dynamic UAV task allocation in uncertain environments, and prioritized higher-value targets and optimizing task execution based on cost-effectiveness. However, all these studies included all UAVs for handling new tasks, which is time-consuming, and the experiments were also conducted on small datasets that do not accurately reflect real-world scenarios. The Task Assignment Method Based on Bionic Algorithms (TABA) method in [52] utilized bionic algorithms (ant colony, bat, and gray wolf) to dynamically allocate tasks among UAVs and plan paths efficiently, but can be criticized for only being tested on small problem instances and for having new task handling strategies that work based on comparisons between the number of UAVs and tasks.

In [53], sequence-to-sequence multi-agent deep deterministic policy gradient (SMADDPG), a reinforcement learning algorithm, was proposed that adapts to changing scales of UAV operations, although its practical testing was limited by the data scale and inefficiencies in managing new tasks. The paper in [54] described an enhanced Multi-Agent Deep Deterministic Policy Gradient (MADDPG) algorithm that works based on deep reinforcement learning and adapts bee colony dynamics for large-scale UAV swarm task allocation, facilitating decentralized decision-making, despite a limitation of not considering the original assignment after detecting new events. The coordinated dynamic task allocation (CDTA) strategy proposed in [55] combines multiagent deep reinforcement learning with a Q-network to dynamically allocate tasks among heterogeneous UAVs, improving the operation speed and reducing the online computational load. However, the proposed approach is restricted to only newly appearing tasks.

### 2.3. Clustering-Based Algorithms

Clustering-based algorithms divide nearby or similar tasks into groups and then assign the groups to UAVs as a cluster assignment, rather than a single task assignment [56]. K-means, one of the most common clustering algorithms, groups similar tasks for UAV assignments, efficiently organizing tasks based on the distances between tasks [57]. Using K-means for clustering, the study in [58] assigned task clusters to the nearest UAV and improved robustness by facilitating task switching among UAVs. However, there was a lack of detail on the task switching and communication methods, which focused solely on task proximity. The paper in [59] integrated auction algorithms with clustering and assigned tasks within clusters to UAVs before seeking additional tasks. This method reduced conflicts but did not detail intercluster communication or consider existing assignments. The research in [60] employed a divide-and-conquer strategy with spectral clustering and PSO to optimize UAV cargo distribution in urban areas, dynamically adjusting to task interruptions. The validation of the above three studies on small-scale problems may not reflect real-world complexity.

The proposed Hybrid Clustering and Partial Reassignment (HCPR) approach addresses key limitations in existing multi-UAV task assignment methods by enhancing scalability, adaptability, and communication efficiency. Unlike traditional clustering methods that only segment tasks, our approach balances the UAV distribution within clusters, ensuring efficient workload management and reducing computational overhead by executing decentralized task allocation locally within each cluster. This eliminates the need for global decision-making, improves parallelism, and reduces energy consumption by minimizing long-distance UAV travel.

Existing decentralized methods suffer from high communication overhead and global reassignment delays when responding to dynamic events. In contrast, our partial reassignment strategy selectively engages only nearby UAVs and releases only the farthest tasks, reducing disruption and improving response time. Additionally, our hybrid communication model optimally restricts intra-cluster communication, while maintaining centralized inter-cluster coordination, minimizing network congestion and improving decision-making efficiency. Furthermore, our proactive idle UAV management mechanism dynamically reallocates idle UAVs to assist overloaded UAVs or transition to uncovered clusters, maximizing resource utilization. By combining clustering, decentralized assignment, selective reassignment, and hybrid communication, HCPR achieves superior scalability, responsiveness, and robustness compared to existing methods, making it highly effective in large-scale, dynamic environments.

## 3. Problem Formulation

The multi-UAV dynamic task assignment problem is an NP-hard combinatorial optimization problem [7,33,57] that can be described as follows: Given a set of *N* UAVs $U=\{U_{1},U_{2},U_{3},\ldots ,U_{N}\}$, each UAV $U_{i}$ is defined by the payload capacity ($L_{i}$), the velocity ($v_{i}$), lidar sensor ($lidar_{i}$), and the assigned tasks list ($a_{i}$), with distinct capabilities for each *U*. The UAVs are heterogeneous, as they differ in payload capacity and velocity, which directly affects their execution time for the same task. A significantly larger set of *M* tasks is also defined as $T=\{T_{1},T_{2},T_{3},\ldots ,T_{M}\}$, where M≫N. Each task $T_{j}$ is characterized by four attributes: start time ($ts_{j}$), end time ($te_{j}$), duration ($D_{j}$), and reward ($R_{j}$). Each task can be performed by, at most, one UAV, and each UAV can perform one task at a time and be assigned to multiple tasks. This specific problem is described as a single-task (ST), single-robot (SR) problem [19]. The mission environment is dynamic and may experience sudden events such as the emergence of new tasks, and failure or idleness of UAVs. The common objectives of the problem are to find an assignment of UAVs to tasks that optimizes the predefined objectives: maximizing the UAV throughput and minimizing the task waiting time costs. Furthermore, when a sudden event occurs, the original assignment is dynamically adjusted, and the UAVs autonomously adapt to these changes. The problem can be mathematically formulated as follows:

(1) $$\begin{array}{cc} \; & \max\sum_{i=1}^{N}\sum_{j=1}^{|a_{i}|}x_{i,j}s_{i,j} \end{array}$$

(2) $$\begin{array}{cc} \; & s_{i,j}=th_{i,j}-c_{i,j} \end{array}$$

(3) $$\begin{array}{cc} \; & c_{i,j}=\tau_{i,j}-ts_{j} \end{array}$$

(4) $$\begin{array}{cc} \; & \tau_{i,j}=\left\{\begin{array}{cc} d_{0,j}/v_{i} & \mathrm{if}\;j=1\\ \tau_{i,j-1}+D_{j-1}+\left(d_{j-1,j}/v_{i}\right) & \mathrm{if}\;1<j\leq |a_{i}| \end{array}\right. \end{array}$$

(5) $$\begin{array}{cc} \; & th_{i,j}=e^{-\lambda c_{i,j}}R_{j} \end{array}$$

**Subject to**

(6a) $$\begin{array}{cc} \; & x_{i,j}\in \left\{0,1\right\} \end{array}$$

(6b) $$\begin{array}{cc} \; & \sum_{j=1}^{|a_{i}|}x_{i,j}\leq L_{i} \end{array}$$

(6c) $$\begin{array}{cc} \; & \sum_{j=1}^{M}\sum_{i=1}^{N}x_{i,j}\leq 1 \end{array}$$

(6d) $$\begin{array}{cc} \; & \tau_{i,j}\in \left[ts_{j},te_{j}\right] \end{array}$$

(6e) $$\begin{array}{cc} \; & \sum_{i=1}^{N}|a_{i}\left|\leq |T|+|nT\right| \end{array}$$

In (1), $s_{i,j}$ is the score value obtained by $U_{i}$ after performing $T_{j}$; and $x_{i,j}$ is a binary variable that is equal to 1 if $T_{j}$ is performed by $U_{i}$, and 0 if $T_{j}$ has expired. The score function given in (2) is based on maximizing the UAV throughput ($th_{i,j}$) and minimizing the tasks costs ($c_{i,j}$) of assigning task $T_{j}$ to UAV $U_{i}$. The tasks costs ($c_{i,j}$) in (3) include the task waiting time from its starting time ($ts_{j}$) until the time ($\tau_{i,j}$) is executed by UAV ($U_{i}$). $\tau_{i,j}$ given in (4) is the time the UAV ($U_{i}$) starts executing task $T_{j}$. If the task is the first task, then $\tau_{i,j}$ will be the distance between the base station and the task location ($d_{0,j}$) over the UAV velocity ($v_{i}$); if it is not the first task, it will be computed based on the execution time of the previous task ($\tau_{i,j-1}$) on the UAV’s tasks list ($a_{i}$), its duration ($D_{j-1}$), and the distance from the previous tasks to the current one ($d_{j-1,j}$) over the UAV’s velocity ($v_{i}$). The UAV throughput ($th_{i,j}$) in (5) represents the total reward from tasks completed by UAVs, adjusted by a time-based decay to emphasize timely execution. This reflects both task completion and responsiveness. The gained throughput after $U_{i}$ performs $T_{j}$ is modeled as an exponential decay function of the waiting time costs and emphasizes the urgency of timely task execution, multiplied by the task initial reward ($R_{j}$). The parameter λ modulates the sensitivity of throughput to delays and encourages quicker task completion.

The objective function is subject to the following constraints: Equation (6a) represents a binary variable that is 1 if $U_{i}$ starts performing $T_{j}$ before its end time $te_{j}$, and 0 if $T_{j}$ expires. Equation (6b) denotes the maximum number of loads $L_{i}$ that can be covered by $U_{i}$. Equation (6c) shows that each task $T_{j}$ can be performed by at most one UAV $U_{i}$. Equation (6d) denotes the time window in which task $T_{j}$ should be executed; $ts_{j}$ is the earliest start time, and $te_{j}$ is the latest start time. Equation (6e) shows that the total number of tasks assigned to all UAVs (*a*) must not exceed the total number of known tasks (*T*) plus the number of new or emergent tasks (nT). The model currently does not incorporate battery limitations, assuming continuous operation (e.g., solar charging). A list of key symbols is provided in Table 1.

**Figure 1:**
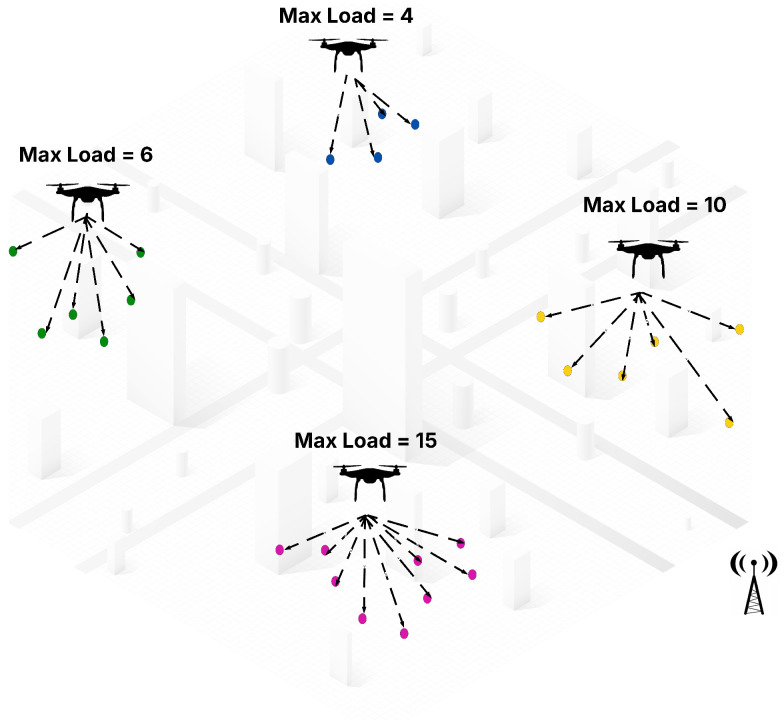
Visual representation of the multi-UAV dynamic task assignment problem. Each UAV is assigned a unique set of tasks (colored dots) and operates under a maximum load constraint. The task allocation ensures that no UAV exceeds its capacity and that each task is assigned to only one UAV.

Figure 1 provides a visual example of the multi-UAV dynamic task assignment problem as formulated in this study. In this scenario, multiple UAVs are deployed within an urban environment, each assigned a distinct set of tasks represented by colored dots. The assignment respects two key constraints: each task is assigned to only one UAV, and each UAV cannot exceed its maximum load capacity, as indicated by the “Max Load” label next to each UAV. This reflects the mathematical constraints defined in Equations (6b) and (6c), which ensure that UAVs operate within their capacity limits and that tasks are uniquely assigned. The spatial distribution of tasks further illustrates the complexity of the problem in terms of travel distance and time constraints, while the centralized antenna symbol hints at the presence of a supervisory controller that facilitates coordination. This diagram helps readers intuitively understand the objective of the problem—to assign tasks to UAVs in a way that maximizes overall mission efficiency, while satisfying operational constraints.

## 4. Proposed Solution

### 4.1. Description

The proposed solution is a hybrid algorithm that integrates clustering-based task segmentation, partial reassignment, and a centralized-distributed communication topology to enhance task allocation in dynamic and large-scale environments. It operates with two main steps: First, a clustering algorithm partitions the operational space into manageable clusters, ensuring a balanced task and UAV distribution, while the task assignment process runs locally within each cluster, without requiring global coordination. Second, a partial reassignment strategy efficiently manages real-time events by selecting only nearby UAVs and releasing the farthest tasks, minimizing disruption and improving responsiveness. Communication remains distributed within clusters for local coordination, while a central controller oversees inter-cluster interactions, optimizing resource utilization and system scalability.

**Figure 2:**
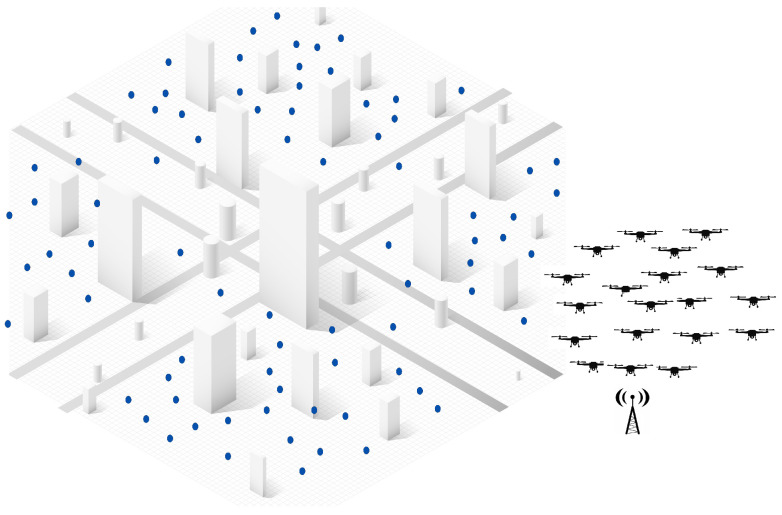
Example of the initial environment setup where UAVs are stationed at the base and tasks are distributed randomly.

Initially, as shown in Figure 2, tasks are scattered throughout the environment, while UAVs remain stationed at the base and wait for deployment upon receiving mission directives. The subsequent paragraphs will go deeper into the two-step approach of the proposed hybrid solution and detail how each phase contributes to optimizing the task allocation process in dynamic and large-scale environments.

**Figure 3:**
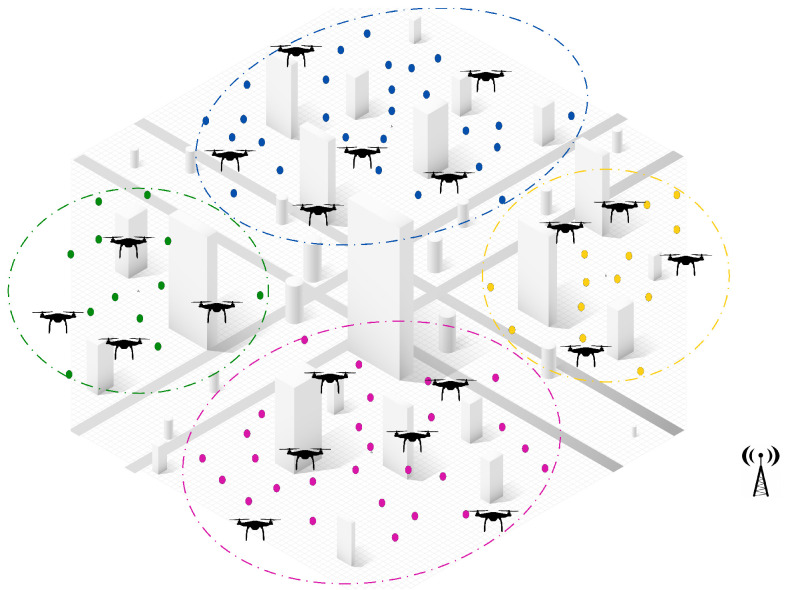
The environment after tasks have been clustered and UAVs have been equally distributed among the clusters.

**Step 1: Task clustering and initial offline assignment.** The first step of the proposed solution involves applying a clustering algorithm to group tasks based on their geographical location and relative distances, ensuring efficient task segmentation. Once the tasks have been clustered, the UAVs are evenly distributed among these clusters by considering workload balance and task density, as illustrated in Figure 3.

Within each cluster, any decentralized assignment algorithm (the CBBA is used in this research) can be employed to manage distributed communication and task allocation among UAVs based on the algorithm’s specific mechanism. The selected assignment algorithm is executed locally within the cluster to establish the initial offline task allocation, without requiring global coordination. At this stage, each UAV maintains its assigned task list and autonomously begins executing its tasks, ensuring efficient resource distribution and independent operation within clusters. This process ensures efficient resource allocation across clusters, giving each task an equal opportunity for early execution, while promoting balanced task urgency management and optimized resource utilization.

**Step 2: Handling Dynamic Events.** In dynamic environments where real-time task assignments are essential, various unexpected events may arise during execution that require rapid and efficient handling. The emergence of new tasks or the potential failure or idleness of a UAV represent the most frequently encountered dynamic changes. The following subsections will illustrate how the proposed solution will effectively address different dynamic scenarios.

**Figure 4:**
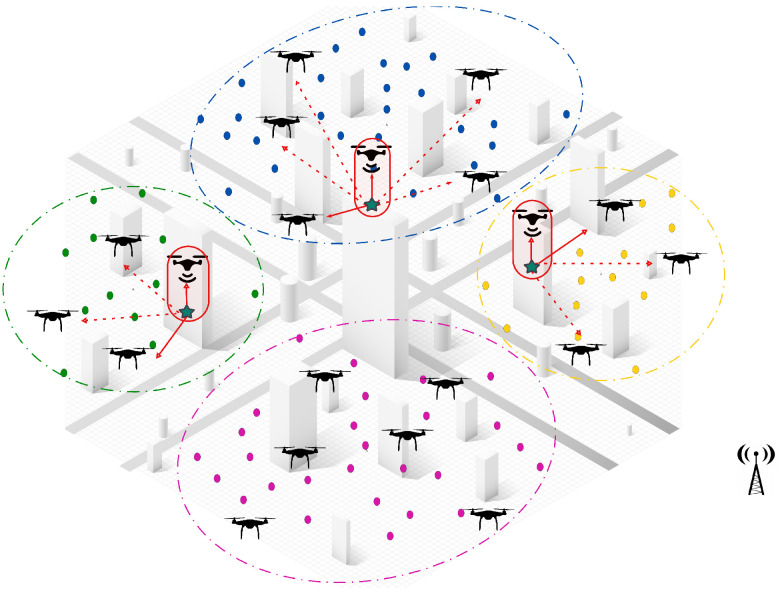
Detection of three new tasks within the green, blue, and yellow clusters, marked by green stars. Distances from these tasks to the cluster’s UAVs are measured locally, with the nearest UAVs selected for the reassignment process, as indicated by the solid red lines.

**Figure 5:**
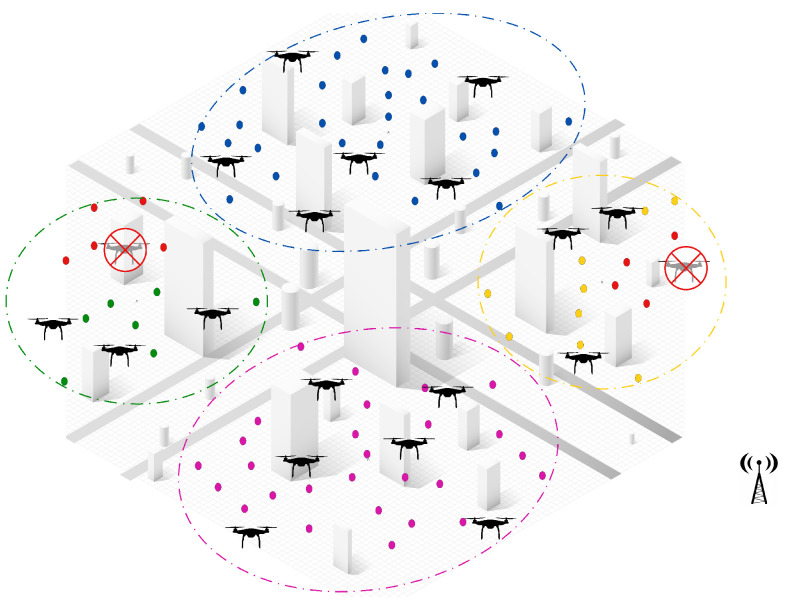
Two UAVs have failed during their missions, one in the green cluster and another in the yellow cluster, with their unperformed tasks marked by red points.

**Figure 6:**
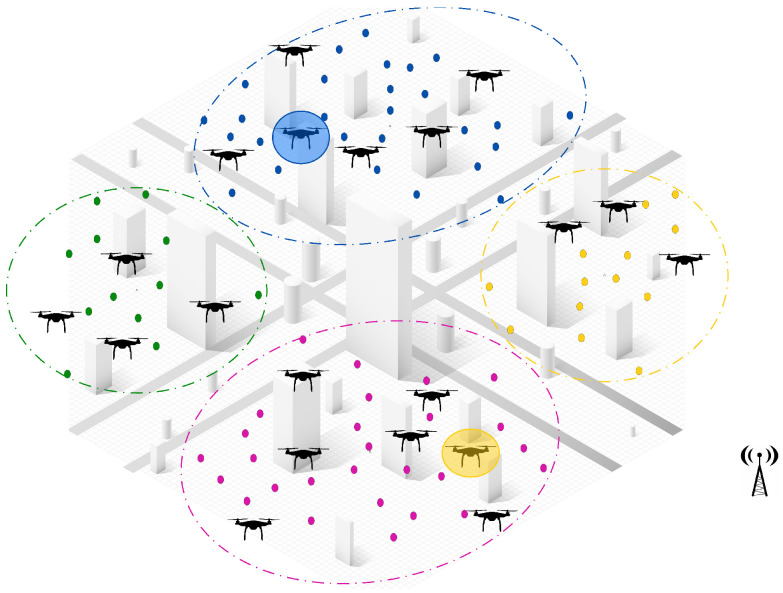
Two UAVs enter an idle state; one in the blue cluster assists the most loaded UAVs within the same cluster, while the other in the yellow cluster migrates to the nearest uncovered cluster, the pink cluster, as the yellow cluster is fully covered.

1. **Appearance of new tasks:** When a new task emerges and is detected by a UAV, the central controller manages the reallocation process among neighboring UAVs within the same cluster. The first step in this process is to check if there are idle UAVs available within the cluster. If an idle UAV is found, it is immediately assigned to the new task without disrupting the ongoing operations. This ensures a quick response to dynamic events, while minimizing the need for task reassignments.
  If no idle UAVs are available, a partial reassignment process is triggered to accommodate the new task. In this step, the central controller selects a sub-team of UAVs that are closest to the newly detected task to participate in the reassignment process. Each selected UAV then releases a subset of its assigned tasks, specifically those that are farthest from its current location and make the lowest contribution to the global objective. By releasing only a portion of their workload, these UAVs free up resources to accommodate the new task without causing a complete reset of assignments, ensuring continuity in ongoing operations.
  Once the tasks have been released, the participating UAVs execute a decentralized CBBA task allocation algorithm locally within the cluster. During this process, the UAVs engage in distributed communication and progressively share and update their decisions until a final allocation has been reached. This localized decision-making ensures rapid task allocation, while avoiding unnecessary global communication overhead.
  Once the decentralized assignment process has converged, the participating UAVs update their task lists and resume their operations. The central controller oversees the process but does not intervene, allowing the UAVs to coordinate and make local decisions, while maintaining efficiency and scalability.
  As illustrated in Figure 4, when three new tasks are detected in different clusters (green, blue, and yellow), the system measures distances between the UAV locations and the detected tasks locally within each cluster. The two nearest UAVs (np = 2) in each cluster, represented by solid red lines, are selected for reassignment. Each UAV then releases two of its farthest assigned tasks (nr = 2), before the locally distributed assignment algorithm reallocates both the released tasks and the new task within the cluster. This approach ensures fast responsiveness to dynamic events, minimizes task disruptions, and optimizes the workload distribution within the cluster.
2. **UAV failure:** In the event of a UAV failure, the central controller initially manages the reassignment process by assessing all unperformed tasks previously assigned to the failed UAV. If an idle UAV is available, the central controller immediately assigns it to take over the tasks, ensuring continuous operation, without triggering a reassignment process. However, if no idle UAV is available, the unperformed tasks are treated as new tasks and integrated into the Partial Reassignment Process, which operates in a distributed manner within the failed UAV’s cluster.
  At this stage, a subset of UAVs closest to the failed UAV’s last known position are selected to participate in the reassignment. These UAVs independently release a portion of their assigned tasks, prioritizing those that contribute the least to the global mission objective or are geographically distant, to accommodate the newly assigned tasks. The distributed CBBA assignment algorithm is then executed locally among the participating UAVs, allowing them to autonomously exchange task availability, workload status, and bid values. This decentralized decision-making ensures that tasks are efficiently reallocated, without requiring intervention from the central controller. By combining centralized intervention for failure detection and initial task management with a distributed reassignment process within clusters, the system demonstrates a hybrid nature, ensuring scalability, responsiveness, and resilience in dynamic environments. Figure 5 shows a failed UAV in the green cluster and another failed UAV in the yellow cluster. All of their unperformed tasks (red points) are considered new tasks.
3. **UAV Idleness:** In a dynamic operational environment, a UAV may enter an idle state after successfully completing all its assigned tasks. To ensure optimal resource utilization and task coverage, the central controller manages idle UAVs through the following steps:
  - Immediate Task Allocation: If a new task is detected or unassigned tasks remain, the central controller immediately assigns the idle UAV to these tasks, ensuring a fast response, without requiring task reassignment from active UAVs.
  - Intra-Cluster Assistance: If there are no new or unassigned tasks but the current cluster still requires coverage, the central controller directs an idle UAV to assist the most heavily loaded UAV in its cluster. This ensures efficient workload balancing within the cluster, optimizing resource utilization, without unnecessary UAV downtime. As shown in Figure 6, the idle UAV in the blue cluster (highlighted circle) is directed by the central controller to support the busiest UAV in the same cluster.
  - Inter-Cluster Migration: If all tasks within the cluster are fully addressed, the central controller directs the idle UAV to migrate to the nearest uncovered cluster that requires additional support. As illustrated in Figure 6, an idle UAV from the yellow cluster moves to the pink cluster, ensuring continuous coverage where UAVs are needed.
  This centralized resource redistribution allows UAVs to maximize task coverage and improve overall mission efficiency in large-scale environments.

### 4.2. Algorithms

The designed algorithms form the foundation of the proposed solution for dynamic task assignment in multi-UAV systems. These algorithms address real-time adaptation to emergent tasks, UAV uncertainties, and efficient utilization of UAVs, and reflect the solution’s ability to maintain robust coordination in real-time scenarios.

In Algorithm 1, the central controller acts as a supervisory entity, ensuring efficient coordination and adaptability of UAV-task assignments. In Step 1 (line 1), the operational environment is initialized, and an offline task assignment is computed to optimize the UAV distribution before execution. In Step 2 (line 2), UAV operations are initiated by the central controller, where each UAV independently executes its assigned tasks in parallel, using mechanisms such as multi-threading, and in a distributed manner, while responding to dynamic events in real time. The central controller does not interfere with task execution but ensures mission continuity by managing dynamic event detection and task reassignment. This hybrid approach integrates centralized mission oversight with decentralized UAV decision-making, ensuring scalability, adaptability, and efficient task execution in dynamic environments.
**Algorithm 1** Central  Controller**Input:** *N*: Number of UAVs. *M*: Number of tasks.*Q*: Number of new tasks.nC: Number of clusters.mpS: Width of the simulation map.dist: Task distribution, which can be random, concentrated, or predetermined.1:**Step 1:** Initialize the environment and compute the initial offline UAV-task assignment by calling Algorithm 2.U← **Algorithm 2** (N,M,Q,nC,mpS,dist)  2:**Step 2:** Initiate UAVs’ operations using a parallel loop, where each UAV executes its assigned tasks simultaneously by calling Algorithm 3.**Parallel for each** $U_{i}\in U$:     $U_{i}\leftarrow$ **Algorithm 3** ($U_{i}$)

Algorithm 2 is a centralized Algorithm that defines the initial offline task assignment process, managed by the central controller. In line 1, a set of tasks and new tasks is generated based on a predetermined distribution model and map size. Each task is characterized by a start time, end time, duration, and reward, and this process is executed by calling Algorithm 4. In line 2, a set of UAVs are initialized with their characteristics, including load, velocity, an empty assigned task list, and a LiDAR sensor, by calling Algorithm 5. In line 3, tasks are grouped into clusters based on geographical proximity using a clustering Algorithm (K-Means is used in this research) to facilitate efficient allocation. In line 4, the UAVs are distributed among clusters in a balanced manner, considering the task density in each cluster and the UAVs’ capacity, by calling Algorithm 6 to optimize resource utilization and ensure an even workload distribution. In line 5, a distributed assignment algorithm (the CBBA is used in this study) is executed locally within each cluster, allowing the UAVs to autonomously coordinate their assignments, while maximizing *s* as defined in Equation (1). This localized execution enhances the efficiency by restricting communication to the participating UAVs within each cluster, reducing unnecessary data exchanges and ensuring scalability in large-scale environments. The algorithm establishes a foundational offline assignment, which can later be dynamically adjusted in response to real-time changes.

Algorithm 3 is a distributed algorithm executed independently by each UAV, enabling real-time adaptability and fully autonomous task execution. Each UAV, at line 1, iterates through its assigned task list ($a_{i}$), performing tasks without requiring external coordination. In lines 2–5, the UAV checks whether a task’s execution window is active. If the task is ready, it navigates to the task location, marks it as active, and begins execution. The execution time is then calculated in line 5 using Equation (4). If a task’s execution window has expired (lines 6–8), the task is marked as inactive. Throughout this process, the UAVs function independently and in parallel, executing assigned tasks without central controller intervention. However, in lines 9–12, if a dynamic event (e.g., a new task appearance, UAV failure, or unexpected environmental change) is detected by the UAV’s LiDAR sensor, the UAV triggers the central controller, which then determines the appropriate response by calling the handleDynamicEvent algorithm. In line 14, once a UAV has completed all assigned tasks and enters an idle state, it notifies the central controller, allowing it to be reassigned to assist other UAVs or cover additional tasks if needed. This approach ensures that, while UAVs function in a fully distributed and autonomous manner, the central controller remains a supervisory entity, intervening only when necessary to maintain mission continuity and task efficiency.
**Algorithm 2** Initial . Offline Tasks-UAVs Assignment**Input**: *N*: Number of UAVs. *M*: Number of tasks.*Q*: Number of new tasks.nC: Number of clusters.mpS: Width of the simulation map.dist: Task distribution, which can be random, concentrated, or predetermined.**Output:** *U*: UAVs after updating their assigned task list upon generating the offline assignment.1:Generate a set of tasks $T=\{T_{1},\ldots ,T_{M}\}$ and set of new tasks $nT=\{nT_{1},\ldots ,nT_{Q}\}$ based on the given distribution dist, by calling Algorithm 4.T← **Algorithm 4** (M,Q,mpS,dist)  2:Define a set of UAVs $U=\{U_{1},\ldots ,U_{N}\}$, by calling Algorithm 5.U← **Algorithm 5** (*N*)  3:Divide the tasks into clusters, using KMean algorithm, based on their geographical locations.C← KMean(T,nC)  4:Distribute UAVs across clusters in a balanced manner, considering each UAV’s capacity load ($L_{i}$) and task density in the respective cluster, by calling Algorithm 6.C← **Algorithm 6** (N,M,nC,C)  5:Assign tasks to UAVs by locally running the Distributed CBBA Algorithm 9 in each cluster to maximize *s* given in Equation (1), then update the assigned task lists ($a_{i}$) for each $U_{i}$.U← **Algorithm 9** (U,T)

Algorithm 4 creates tasks for UAV simulations based on specified parameters. It begins by initializing tasks that exist and are known before the mission starts (Lines 1–4). For each task (from 1 to M), it generates spatial coordinates ($x_{j},y_{j},z_{j}$) based on the width of the simulation map (mpS) and a defined distribution (dist). Subsequently, each task $T_{j}$ is assigned random values for arrival time ($ts_{j}$), end time ($te_{j}$), duration ($D_{j}$), and reward ($R_{j}$). The process is repeated similarly for a set of new tasks that dynamically appear during execution (nT), enumerated from 1 to *Q* (Lines 5–8). For each new task $nT_{q}$, it generates spatial coordinates and assigns random values for arrival time, end time, duration, and reward, mirroring the procedure used for existing tasks. The information of new tasks is then set globally, at line 9, for any further detection or processing by the controller or UAVs. This structured approach ensures that both sets of tasks are equipped with all necessary attributes for further simulation and task assignment processes.

Algorithm 5 is designed to set up the UAVs with the necessary configurations for operational readiness. The process begins by iterating over a predefined number of UAVs, *N* (Lines 1–5). For each UAV, denoted as $U_{i}$, the algorithm first sets its start location to the coordinates of a base station (Line 2). Subsequently, it initializes each UAV’s maximum load capacity ($L_{i}$) and velocity ($v_{i}$) with predefined values (Line 3), preparing them for task handling and movement according to the operational requirements. Finally, each UAV is equipped with a LiDAR sensor (Line 4), enhancing its capability to detect and respond to environmental variables. This setup process equips each UAV with the essential tools and information needed for effective deployment across various clusters, ensuring they are ready for further task assignments and operations.
**Algorithm 3** Fully . Distributed Dynamic Task Execution**Input:** $U_{i}$: UAV information, including assigned task list ($a_{i}$), capacity load ($L_{i}$), and velocity ($v_{i}$).**Output:** $U_{i}$: Updated UAV information after task execution. 1:**for** $j=1\;\mathrm{To}\;\left|a_{i}\right|$ **do** 2:    **if** $ts_{j}\leq \mathrm{currentTime}\mathrm{and}te_{j}\geq \mathrm{currentTime}$ **then** 3:        Move $U_{i}$ to $T_{j}$ location. 4:        Set $x_{i,j}=1$. 5:        Compute $\tau_{i,j}$ based on Equation (4). 6:    **else if** $te_{j}<\mathrm{currentTime}$ **then** 7:        Set $x_{i,j}=0$. 8:    **end if**  9:    Read $U_{i}$ lidar sensor.     lidarReading← read($lidar_{i}$) 10:    **if** $U_{i}$ detects a dynamic event **then**11:        Notify the central controller of the event occurrence by calling Algorithm 7.          (U←Algorithm7(i,U,lidarReading))12:    **end if**13:**end for** 14:Notify the central controller of the idleness state by calling Algorithm 7.(U←Algorithm7(i,U,UAVidleness))

**Algorithm 4** Generate  Tasks
**Input:**

*M*: Number of existing tasks to be generated.*Q*: Number of new tasks to be generated.mpS: Width of the simulation map.dist: Task distribution across the map, which can be random, concentrated, or predetermined.
**Output:**

*T*: Set of defined tasks, including their coordinates, arrival time, reward, and duration.

1:**for** j=1 **to** *M* **do**2:    Generate initial coordinates $\left(x_{j},y_{j},z_{j}\right)$ for $T_{j}$ based on mpS and dist.    $coord_{j}\leftarrow \left(x_{j},y_{j},z_{j}\right)$.3:    Generate arrival time, end time, duration, and reward for $T_{j}$:     $[ts_{j},te_{j}]\leftarrow$ Random arrival time and end time.    $D_{j}\leftarrow$ Random duration.    $R_{j}\leftarrow$ Random reward.4:**end for** 5:**for** q=1 **to** *Q* **do**6:    Generate initial coordinates $\left(x_{q},y_{q},z_{q}\right)$ for $nT_{q}$ based on mpS and dist.     $coord_{q}\leftarrow \left(x_{q},y_{q},z_{q}\right)$.7:    Generate arrival time, end time, duration, and reward for $nT_{q}$:     $[ts_{q},te_{q}]\leftarrow$ Random arrival time and end time.     $D_{q}\leftarrow$ Random duration.     $R_{q}\leftarrow$ Random reward.8:
**end for**
9:Set nT as global data.


Algorithm 6 systematically distributes the UAVs among the various clusters to ensure an even workload distribution based on the number of tasks in each cluster. In step 1, the algorithm starts at line 1 by calculating the average number of tasks each UAV should handle, given by dividing the total number of existing tasks (*M*) by the total number of UAVs (*N*). It initializes a count of unassigned UAVs as *N* (line 2). For each cluster (from 1 to *n*C, the total number of clusters), it first determines the number of tasks within that cluster and then computes the initial number of UAVs needed for that cluster based on the average task load per UAV (lines 4–5). The count of unassigned UAVs is updated at line 6 after allocating UAVs to each cluster. In step 2, the algorithm checks if there are unassigned UAVs remaining after the initial distribution, the algorithm seeks to allocate these extra UAVs to clusters based on need (line 8). It identifies the most crowded cluster, i.e., the cluster with the highest initial UAV load (lines 10–15). For each unassigned UAV, it assigns it to this cluster, thereby increasing the UAV count for that cluster and ensuring a more balanced distribution of UAVs across tasks. After all UAVs have been allocated, each cluster is assigned its respective subset of UAVs based on the final calculated needs (Line 20).
**Algorithm 5** Initialize UAVs and Sensors**Input:***N*: Number of UAVs.**Output:***U*: UAVs’ current information, including assigned task list, velocity, load and current location.1:**for** i=1 **to** *N* **do**2:    Initialize the assigned task list of $U_{i}$ as empty and set its location to the station’s coordinates.     $a_{i}\leftarrow \phi$.     $loc_{i}\leftarrow$ Station’s coordinates.  3:    Initialize $U_{i}$ maximum load and velocity.     $L_{i}\leftarrow$ Given max load value.     $v_{i}\leftarrow$ Given velocity value.  4:    Mount $U_{i}$ platform with a Lidar sensor for detection purposes.     ${\mathrm{lidar}}_{i}\leftarrow$ Mount(LidarSensor).5:**end for**

Algorithm 7 manages dynamic events occurring during UAV operations, ensuring robustness in task allocation and execution. When a UAV detects a dynamic event in Algorithm 3, it triggers a notification to the central controller, which then initiates Algorithm 7 to analyze the event type and determine the appropriate action. If the event is a new task appearance (lines 1–4), the central controller captures the details of the new task and checks for available idle UAVs. If an idle UAV is found, it is immediately assigned to the task. Otherwise, the controller invokes the Partial Reassignment Process (Algorithm 8), which integrates the new task into the existing framework, while minimizing disruption.

If the event involves a UAV failure (lines 5–9), the central controller first identifies all unperformed tasks assigned to the failed UAV and removes it from the operational pool. If an idle UAV exists in the same cluster, the controller immediately reassigns the unperformed tasks to it, minimizing mission disruption. Otherwise, these tasks are handled similarly to newly detected tasks and are reassigned using the Partial Reassignment Process. This ensures an optimized workload distribution and uninterrupted mission execution.

For UAV idleness detection (lines 10–18), the central controller checks for unassigned or newly detected tasks. If available, an idle UAV is immediately assigned to those tasks. If no tasks remain, the UAV assists the most overloaded UAV within the same cluster, promoting cooperative task execution. If all tasks within the cluster have been completed, the controller redirects the idle UAV to a neighboring cluster requiring assistance, ensuring efficient resource utilization and sustained mission continuity. By continuously monitoring the mission environment, the central controller supervises dynamic event handling, ensuring efficient UAV coordination, minimal disruption, and optimized task execution across clusters.

Algorithm 8, termed the “Partial Reassignment Process”, operates using both centralized and distributed mechanisms, ensuring an efficient and adaptive task reassignment strategy. The process begins with a centralized step, where the central controller identifies the nearest (np) UAVs to the newly detected task newTasks and determines the farthest (nr) tasks from each of these UAVs, to be released for reassignment (line 1). This ensures that the reassignment process is initiated in an optimized manner, selecting UAVs and tasks that minimize disruption, while maximizing efficiency. Once the participants in the reassignment process have been determined, each selected UAV releases the farthest tasks from its assigned task list $a_{k}$ (lines 2–4), and these released tasks, along with the new task, are combined into a reassignment pool (line 5).
**Algorithm 6** Balance UAVs Across Clusters**Input:***N*: Total number of available UAVs.*M*: Total number of existing tasks.nC: Number of clusters.*C*: Set of clusters, where each contains a subset of tasks.**Output:***C*: Clusters after distributing UAVs among them.**Step 1: Compute the Initial Number of UAVs Per Cluster** 1:Compute the initial number of tasks per UAV:nTU←⌊M/N⌋ 2:Initialize the number of unassigned UAVs:unU←N 3:**for** k=1 **to** nC **do** 4:    Calculate the number of tasks in cluster $C_{k}$:     $nTC_{k}\leftarrow \left|C_{k}\right|$ 5:    Compute the initial number of UAVs for cluster $C_{k}$:     $nUC_{k}\leftarrow \left\lfloor nTC_{k}/nTU\right\rfloor$ 6:    Update the number of unassigned UAVs:     $unU\leftarrow unU-nUC_{k}$ 7:**end for****Step 2: Balance and Distribute Extra UAVs Among Clusters** 8:**if** unU≠0 **then** 9:    **for**  q=1 **to** unU **do**10:        Initialize the most crowded cluster:        idxCrowdedC←0        lCrowdedC←011:        **for** k=1 **to** nC **do**12:           Compute initial UAV load in cluster $C_{k}$:            $IUL_{k}\leftarrow \left\lfloor nTC_{k}/nUC_{k}\right\rfloor$13:           **if** $IUL_{k}>lCrowdedC$ **then**14:               Update the most crowded cluster index and load.               $lCrowdedC\leftarrow IUL_{k}$               idxCrowdedC←k15:           **end if**16:        **end for**17:        Assign the extra UAV to the most crowded cluster.         $nUC_{idxCrowdedC}\leftarrow nUC_{idxCrowdedC}+1$18:    **end for**19:**end if**20:Assign UAVs to each cluster based on $nUC_{k}$.

The process is then transitioned to a fully distributed operation. At this stage, the chosen distributed assignment algorithm (the CBBA is used in this study) is executed locally among the participating UAVs, their released tasks, and the newly detected task, to determine the final assignment (line 6). The assignment follows the mechanism of the selected algorithm, where UAVs communicate in a restricted, localized manner, exchanging relevant information (e.g., bids, priorities, or cost evaluations) to collaboratively determine the task allocation result. To reduce communication overhead, the CBBA-based reassignment is limited to a subset of UAVs affected by dynamic changes, and bid exchanges are restricted to local neighbors. This selective and localized exchange significantly lowers the total communication burden compared to a fully distributed reassignment involving all UAVs. This approach ensures that task reassignment is performed without requiring global coordination, enhancing scalability and decentralized cooperation.
**Algorithm 7** Dynamic Event Handler**Input:***i*: Index of the UAV that detected a dynamic event.U: UAVs’ information, including assigned task lists (*a*), capacity loads (*L*), and velocities (*v*).lidarReading: Information about the dynamic event.**Output:**U: Updated UAVs’ task assignments. 1:**if** *lidarReading == Detecting a new Task* **then** 2:    newTasks← Newly detected task(s) by $U_{i}$ 3:    Assign newTasks to idle UAVs if available; otherwise, invoke Algorithm 8 for reassignment.     (U←Algorithm8(i,U,newTasks)) 4:**end if**  5:**if** *lidarReading == UAV failure* **then** 6:    newTasks← Unperformed tasks from $a_{i}$ 7:    Remove $U_{i}$ from *U* 8:    Assign newTasks to idle UAVs if available; otherwise, invoke Algorithm 8 for reassignment.     (U←Algorithm8(i,U,newTasks)) 9:**end if** 10:**if** *lidarReading == UAV idleness* **then**               ▹$a_{i}=\emptyset$11:    **if** Unassigned or newly detected tasks exist **then**12:        Assign available tasks to $U_{i}$.13:    **else if** All tasks are assigned but some UAVs are overloaded **then**14:        $U_{i}$ assists the most loaded UAV in the same cluster.15:    **else**16:        Direct $U_{i}$ to assist the nearest uncovered cluster.17:    **end if**18:**end if**

Finally, in line 7, the assigned task lists of the participating UAVs are updated, integrating the newly detected task, while redistributing the released tasks among the UAVs, and their operations are reinitiated by calling Algorithm 3. By combining a centralized selection of participants and a distributed execution of task reassignment, this algorithm demonstrates the hybrid nature of the proposed system, leveraging the strengths of centralized supervision for structured decision-making and distributed execution for efficient, autonomous UAV coordination.
**Algorithm 8** Partial Reassignment Process**Input:***i*: UAV that detected the dynamic event.U: UAVs’ information, including assigned task lists (*a*), capacity loads (*L*), and velocities (*v*).newTasks: Newly detected task.**Output:**U: Updated UAVs’ task assignments.1:pU←IdentifytopnpUAVsthesameclusterwiththesmallestdistancetonewTask  2:**for** each $U_{k}\in pU$ **do**3:    ${\mathrm{releasedTasks}}_{k}\leftarrow \;\mathrm{Release}\;\mathrm{top}\;nr\;\mathrm{tasks},\;\mathrm{in}\;a_{k},\;\mathrm{with}\;\mathrm{the}\;\mathrm{farthest}\;\mathrm{distance}\;\mathrm{from}\;U_{k}$4:**end for** 5:Combine newTasks with the released tasks into reassignedTasks.reassignedTasks←releasedTasks+newTasks6:Re-run the distributed CBBA Algorithm by calling Algorithm 9 with the selected np UAVs and reassignedTasks.pU← **Algorithm 9** (pU,reassignedTasks) 7:Update task lists (*a*) of the np UAVs and re-initiate their operation by calling **Algorithm 3**.

In Algorithm 9, the distributed task assignment process begins in line 1, where a set of UAV parameters are initialized. Although initial assignments are first performed in Algorithm 5, the CBBA process requires each UAV to independently initialize its local data structures, including bid values, bundle lists, and winner lists. This is essential for enabling decentralized decision-making and conflict resolution during the consensus phase.
**Algorithm 9** Consensus-Based Bundle Algorithm (CBBA) for Distributed Task Assignment**Input:***T*: Tasks information, including their start time ($ts_{j}$), end time ($te_{j}$), duration ($D_{j}$), and reward ($R_{j}$).*U*: UAVs information, including their assigned tasks lists (*a*), capacity load (*L*) and velocity (*v*).**Output:***U*: Updated UAVs’ information. 1:**Initialization:** For each $U_{i}\in U$, initialize $a_{i}\leftarrow \emptyset$, bid list $W_{i}\leftarrow 0$, and winning UAV list $B_{i}\leftarrow -1$. 2:**while** Tasks remain unassigned **do** 3:    **for** each $U_{i}\in U$ **do**             ▹ **Bundle Construction (Task Bidding)** 4:        Compute $s_{i,j}$ given in Equation (1) for each $T_{j}\in T$ 5:        Select the task $T_{j}$ maximizing $s_{i,j}$ while satisfying constraints 6:        **if** $T_{j}$ is feasible **then** 7:           Add $T_{j}$ to $a_{i}$, update $W_{i}\left[j\right]$, and set $B_{i}\left[j\right]\leftarrow i$ 8:        **end if** 9:    **end for** 10:    **for** each $U_{i}\in U$ **do**             ▹ **Consensus Phase (Conflict Resolution)**11:        Exchange $W_{i}$ and $B_{i}$ with neighbors12:        **for** each $T_{j}\in a_{i}$ **do**13:           **if** another UAV $U_{k}$ has $W_{k}\left[j\right]>W_{i}\left[j\right]$ **then**14:               Remove $T_{j}$ from $a_{i}$, update $B_{i}\left[j\right]\leftarrow k$15:           **end if**16:        **end for**17:    **end for**18:**end while**

The algorithm progresses to lines 2–9, where the bundle construction phase (task bidding) is performed. In line 4, each UAV evaluates all available tasks and computes a bid value based on the objective function (1), which reflects the UAV’s suitability for the task based on proximity, timing, and task reward. Each UAV maintains an internal view of task availability, considering only tasks it can feasibly perform given its current position and remaining load capacity. In line 5, the UAV selects the most valuable task that satisfies the constraints and updates its bid and assignment lists in line 7. These lists represent both the UAV’s planned task sequence and the associated bid values for each task. In lines 10–18, the consensus phase (conflict resolution) is initiated. In line 11, UAVs exchange three key elements of information with their neighbors: (1) task availability (i.e., which tasks they are bidding for), (2) current workload status (number of tasks and capacity usage), and (3) bid values associated with their selected tasks. If multiple UAVs bid on the same task, the UAV with the highest bid retains the task, while the others remove it from their assignment lists (lines 13 and 14). This iterative consensus process ensures conflict-free and efficient task distribution across the UAV team. Finally, the process iterates until all tasks have been assigned or no further bids can be made, and the Algorithm returns the updated UAV task allocations. Although the bid and winner information is exchanged only among neighboring UAVs, the CBBA ensures global convergence through the iterative propagation of local updates. When two non-adjacent UAVs bid on the same task, the conflict is resolved through their mutual neighbors, who relay updated bid values and task ownership information. The algorithm converges when no UAV modifies its bid or winner list in the subsequent rounds, indicating that a stable consensus has been reached. This decentralized execution enables UAVs to efficiently coordinate their assignments in a fully distributed manner, without a central controller.

### 4.3. Novelty of the Proposed Solution

The proposed solution addresses three key challenges in the existing state-of-the-art literature. First, it aims to reduce the communication burden and minimize delays between UAVs. Second, it enhances robustness against changes in environmental dynamics. Third, it addresses the challenge of dealing with large-scale problem instances. Subsequent sections will provide detailed insights into how the proposed solution effectively addresses each of these problems.

**Figure 7:**
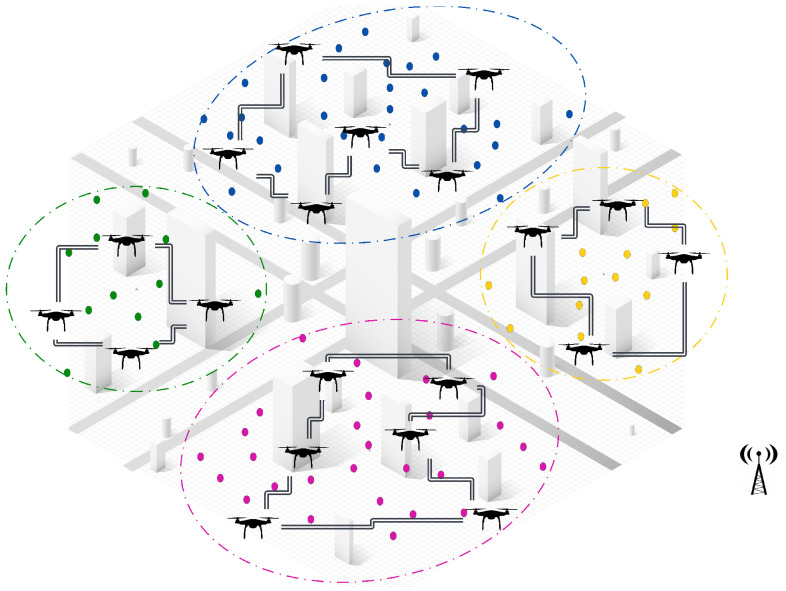
Communication within the clusters is limited under scenario (a) to interactions between UAVs before the mission starts, for collecting offline assignment results, and then they operate autonomously.

**Figure 8:**
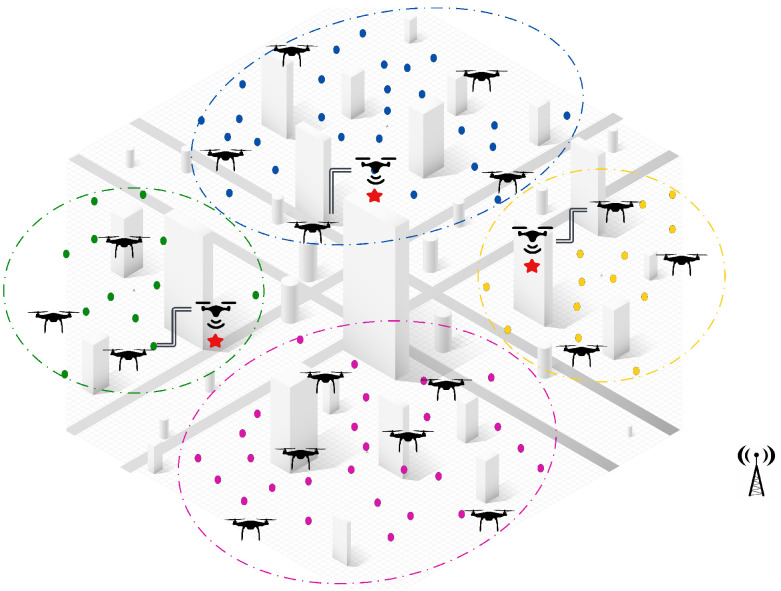
Communication within the clusters is limited to a specific sub-team of UAVs, activated only when dynamic events are detected and the partial reassignment process is initiated.

**Figure 9:**
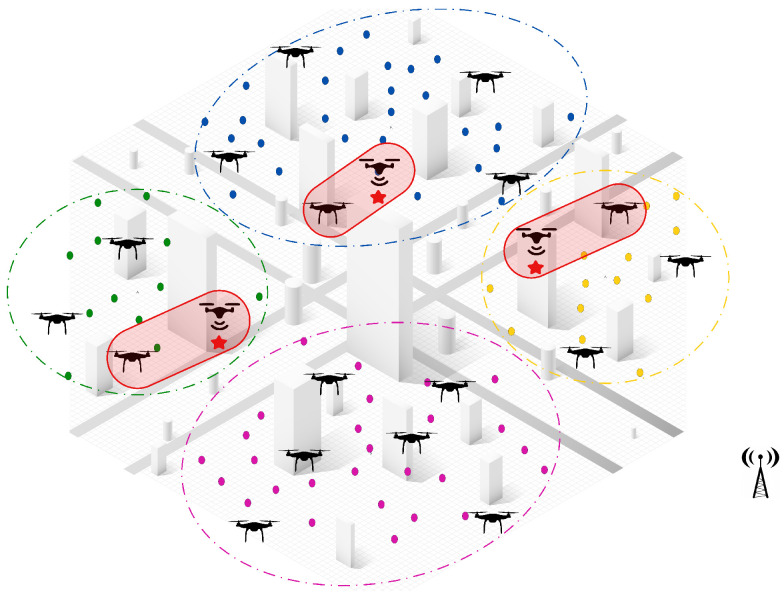
The partial reassignment process, triggered by the detection of a new task, is limited to a small subgroup of UAVs that are geographically closest to the new task.

1. **Communication Bandwidth:** As a dynamic assignment problem, the interaction and information sharing among UAVs must be rapid and restricted. Therefore, the proposed solution establishes a centralized-distributed communication topology connecting UAVs both within and between clusters. This approach serves to reduce communication overhead, reduce delay, and ultimately enhance overall performance.
  - *Communication Inside Clusters:* The proposed solution employs a distributed communication strategy within clusters to ensure efficient task allocation and minimal network overhead. This occurs in two decentralized scenarios, where a distributed CBBA assignment algorithm is executed autonomously by the UAVs.
    (a) First, before the mission starts, the UAVs within each cluster run the distributed assignment algorithm to generate the offline task allocation. The UAVs communicate locally within their cluster, following the mechanism of the selected distributed algorithm to determine task assignments. Once the allocation has been finalized, the UAVs operate autonomously and independently, without requiring further coordination, as shown in Figure 7.
    (b) Second, when a dynamic event occurs (e.g., new task, UAV failure), the central controller selects the nearest UAVs, which then execute the distributed CBBA assignment algorithm to reassign both the new and released tasks. Only participating UAVs communicate within their cluster, keeping the process of task reassignment fully decentralized and efficient, as shown in Figure 8.
  - *Communication Among Clusters:* Beyond the decentralized task execution within clusters, the system remains centralized for mission supervision and inter-cluster coordination, where the central controller oversees operations and triggers reassignment when necessary. This hybrid approach ensures scalability, efficiency, and adaptability by combining decentralized UAV autonomy with centralized decision-making. By restricting inter-cluster communication to the central controller, the system reduces unnecessary UAV-to-UAV communication overhead, enhancing coordination, resource utilization, and overall operational efficiency.
2. **Handling Dynamic Events:** Efficiently managing dynamic events is a critical aspect of our proposed hybrid solution, which is designed to handle the complexities of real-time changes within multi-UAV operations. Upon the detection of a dynamic event, the proposed solution does not require the participation of all UAVs. Instead, it selectively engages a subteam of UAVs that are geographically closest to the new event. This partial participation strategy reduces the disruption to the fleet and maintains ongoing task performance, while swiftly incorporating the new task into the allocation. Furthermore, the proposed solution incorporates a task release mechanism whereby only a portion of the previously assigned tasks are released during reallocation. This approach is particularly useful when integrating new tasks or redistributing the load due to a UAV failure. By releasing only tasks that are less critical or farther from the UAVs’ current positions, we minimize the impact on overall mission performance and focus resources on time-sensitive tasks. Overall, this strategy enhances the responsiveness of the UAV fleet to dynamic events, strengthens the robustness of the solution, and adapts rapidly and effectively to changing operational conditions, without significant disruptions (Figure 9).
3. **Large-scale Problem Instances:** The proposed solution excels in managing large-scale problem instances, which are particularly challenging due to the extensive coordination and computation needed. By employing a sophisticated clustering approach, the algorithm segments the operational area into smaller manageable units, each handled by a subset of UAVs. Clustering ensures a more organized deployment and optimizes the coverage and responsiveness of the UAV network, which will be highly effective in extensive and complex scenarios. By focusing on local clusters, while maintaining a global overview through centralized control, our solution achieves a balanced distribution of tasks, optimal resource usage, and a significant reduction in operational delays, which are critical for scalability and efficiency in large-scale applications.

## 5. Empirical Evaluation

### 5.1. Simulation Setup

The implementation environment for the proposed solution was based on MATLAB 2023b coupled with the UAV Toolbox, which provides a comprehensive suite of tools for UAV mission planning and simulation. Additionally, the parallel computing toolbox was utilized to address computationally and data-intensive challenges by leveraging multicore processors that parallelize extensive experiments and provide significantly better performance. The experiments were conducted using high-specification resources from Amazon Web Services (AWS), which offers a broad set of global cloud-based products and includes computing units (CPUs) and memory storage. We used Amazon EC2 C6a instances, which supports increased sizes of up to 192 vCPUs per instance and 384 GB of memory. These instance features are designed for computationally intensive workloads.

### 5.2. Evaluating the Performance of the HCPR Algorithm

This experiment aimed to test the objectives and evaluate the effectiveness of the HCPR algorithm across different dataset sizes and complexity levels. The study encompassed two experimental scales: small and large, as presented in Table 2. The predefined parameter settings for the various scenarios are presented in Table 3. Additionally, in Equation (5), the time decay parameter λ was set to 0.05 to moderately penalize delayed task execution, and task rewards $R_{j}$ were randomly generated within the range [30, 100] to reflect varying task priorities. The effectiveness of the HCPR algorithm across different dataset sizes was compared with that of the consensus-based bundle algorithm (CBBA), which is a decentralized approach to task allocation in multiagent systems. The algorithm operates through two main phases: the bundle construction phase, where each UAV creates a list of desired tasks based on local greedy optimization, and the consensus phase, where UAVs communicate to resolve conflicts and agree on task assignments. When a new task appears, the CBBA triggers a full reassignment process where all UAVs release their current task assignments. In the event of UAV failure or when a UAV becomes idle, the CBBA currently takes no action. Each scenario was iteratively run 10 times for a total of 2500 runs (1250 runs for each scale). Statistical analysis then involved computing the mean and standard deviation for the outcomes of each scenario. As outlined in the Introduction section, the proposed solution has three main objectives that were evaluated and proven using specific evaluation metrics:

1. Maximize Responsiveness: This objective ensures that the proposed solution reacts quickly and effectively to start and complete mission tasks. Metrics such as task waiting time and the number of performed tasks were used to assess this responsiveness.
2. Increase Scalability: Scalability involves the solution’s ability to manage an increasing number of tasks or to operate effectively across broader geographic areas, without performance degradation. This was measured using UAV throughput and mission completion time under varying instance sizes.
3. Improve Solution Robustness: This objective assesses the solution’s ability to adapt to unexpected operational changes, such as new tasks or UAV failures, while maintaining consistent performance. Robustness was quantified by the number of detected and covered dynamic tasks.

To assess the achievement of the solution’s objectives, five metrics were used:

1. Waiting Time: Time elapsed before a UAV begins a task.
2. UAV Throughput: Total reward for tasks completed by UAVs, adjusted for time discounting, where the rewards decrease with late task execution times.
3. Number of Performance Tasks: Total number of tasks completed by the UAV fleet.
4. Number of Covered New Tasks: Number of new tasks detected and performed during the mission.
5. Completion Time: Total time taken to complete all allocated tasks.

#### Result Analysis

**Figure 10:**
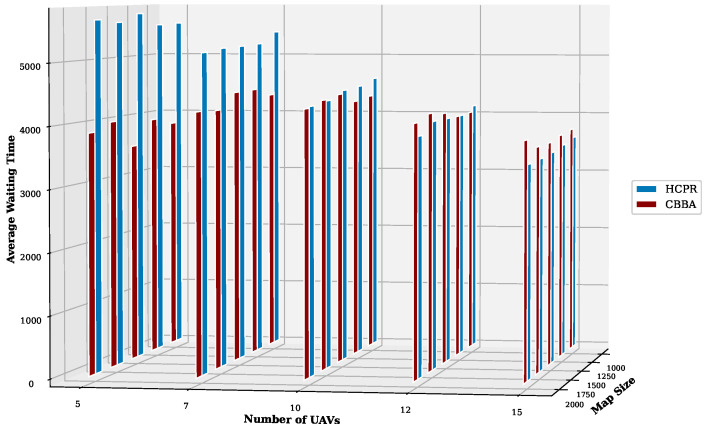
Average waiting time for small instances.

**Figure 11:**
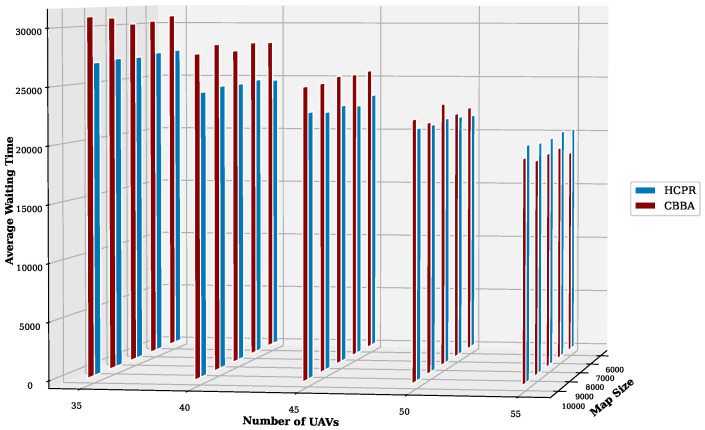
Average waiting time for large instances.

**Figure 12:**
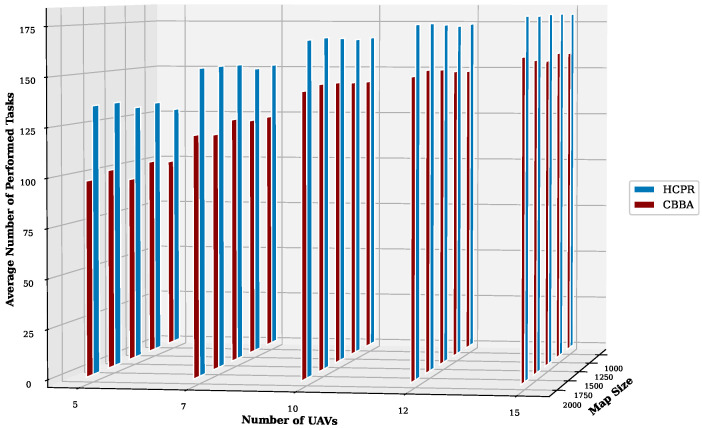
Average number of performed tasks for small instances.

**Figure 13:**
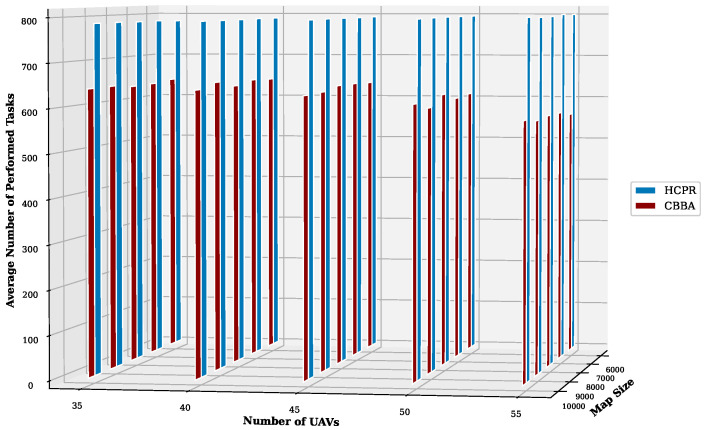
Average number of performed tasks for large instances.

**Figure 14:**
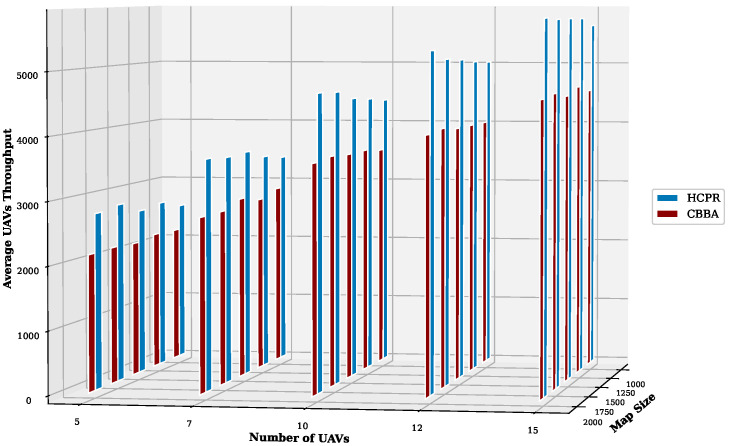
Average UAV throughput for small instances.

**Figure 15:**
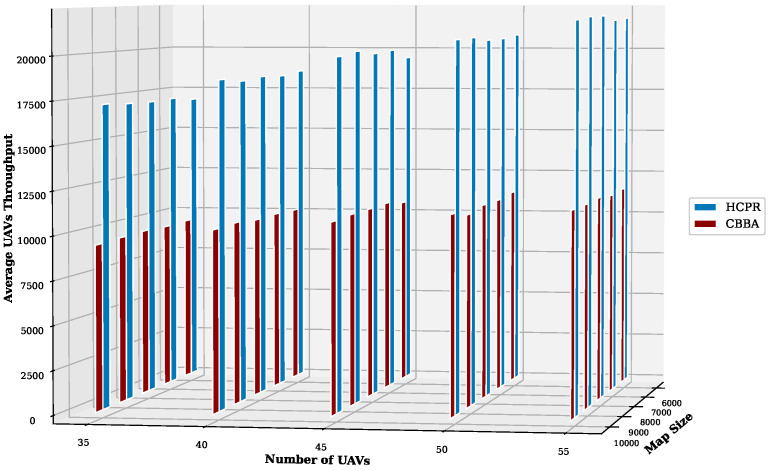
Average UAV throughput for large instances.

**Figure 16:**
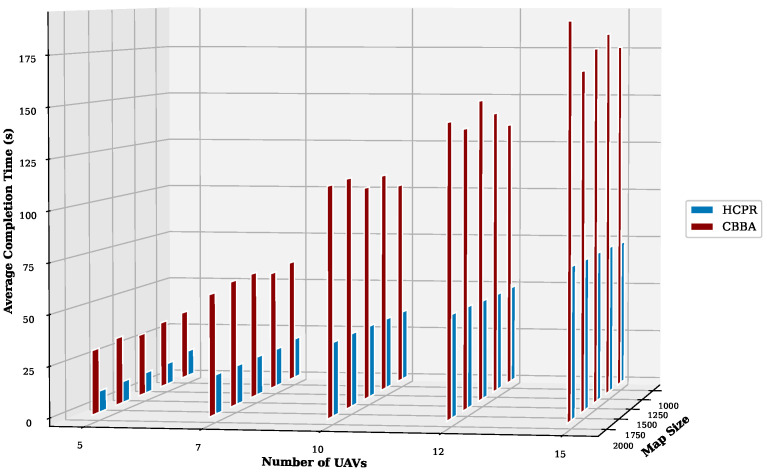
Average completion time for small instances.

**Figure 17:**
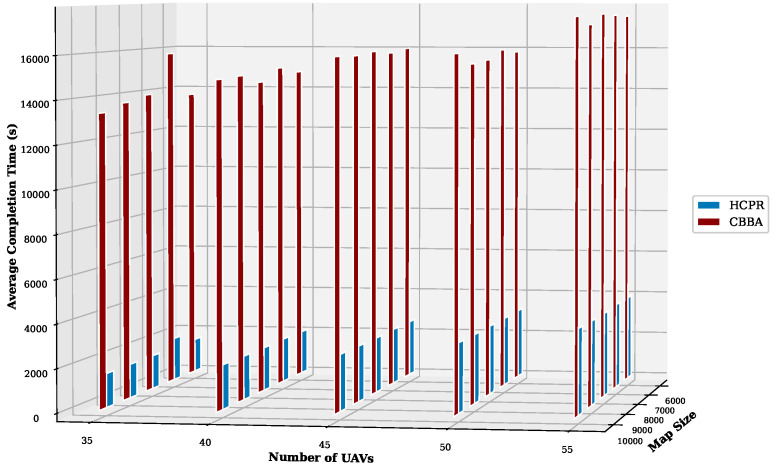
Average completion time for large instances.

**Figure 18:**
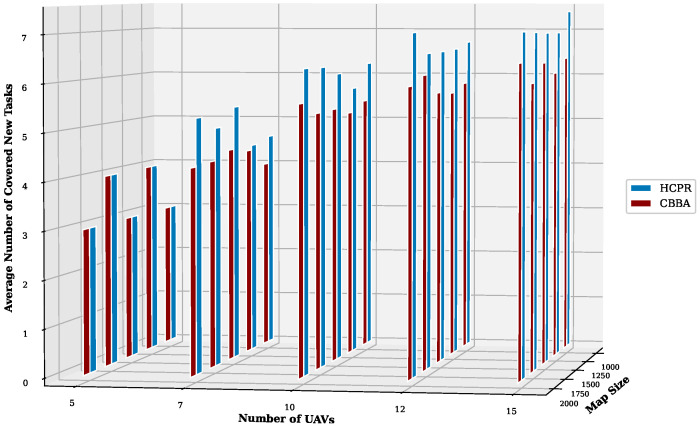
Average number of detected new tasks for small instances.

**Figure 19:**
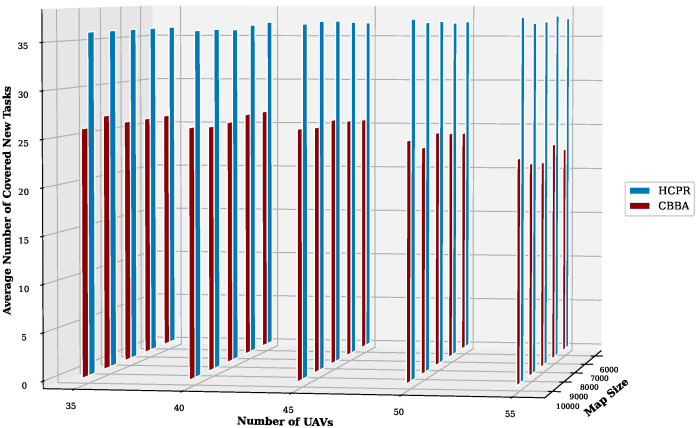
Average number of detected new tasks for large instances.

1. **Solution Responsiveness:** We evaluated the solution’s responsiveness using the waiting time and the number of performed tasks metrics, where the waiting time reflects the speed at which UAVs begin performing tasks and the number of performed tasks indicates the solution’s efficiency in handling tasks within specific time frames and underscores its reactive capabilities.
  (a) **Average Task Waiting Time:**
    As shown in Figure 10, on the small dataset, the CBBA generally exhibited lower average waiting times when the number of UAVs was on the lower end (5 to 7 UAVs). However, as the number of UAVs increased, the performance of HCPR relative to the CBBA improved, particularly at higher UAV counts (12 and 15). This indicates that HCPR’s response improved with a larger number of UAVs. An increase in the map size did not have a consistent impact on the performance of either algorithm, since the UAV velocity is proportional to the map size, as described above. For the large dataset (Figure 11), HCPR consistently showed lower average waiting times across almost all scenarios, particularly as the number of UAVs increased. HCPR demonstrated remarkable responsiveness in large-scale scenarios, and improved its performance advantage as the scale of operations increased. To justify and analyze how HCPR outperformed the CBBA, several reasons can explain the enhanced efficiency and reduced waiting times observed in Figure 10 and Figure 11:
    - Geographical Distribution of UAVs: Distributing UAVs among different clusters allows tasks throughout the area to have an equal opportunity for earlier execution, thereby reducing their waiting times. In contrast, with the CBBA, where UAVs are concentrated around the base station, tasks located in distant areas experience longer waiting times and may expire before being reached by any UAV.
    - Handling Dynamic Events with Nearby UAVs: Assigning a small team of nearby UAVs to address dynamic events can significantly reduce task waiting times. This approach speeds up the reassignment process by involving only a limited number of UAVs and selecting those closest to the event and minimizing travel distances. Furthermore, by partially releasing only a small set of the farthest tasks for the reassignment process, this strategy shortens the reassignment duration and allows new tasks, which are nearer to the detected UAVs, to be executed sooner, with reduced waiting times. It also gives these distant tasks an opportunity to be reassigned to UAVs that are closer, potentially also accelerating their execution. This also ensures that closer original tasks maintain their scheduled earlier execution times. Conversely, the CBBA’s method of reallocating all tasks and involving all UAVs leads to work interruptions and extended delays in task execution, thereby increasing waiting times.
    - Utilization of Idle UAVs: Employing idle UAVs to handle new tasks or assist others within the same or neighboring clusters enhances the speed of task execution and reduces waiting times, ensuring a more efficient use of available resources.
    - Communication Restriction: Limiting communication to only involved UAVs and central control within and between clusters helps maintain task execution, without delays caused by dynamic events. This approach allows most UAVs to continue their tasks uninterrupted and leads to earlier completion and reduced waiting times. In the CBBA, however, the requirement for all UAVs to communicate after detecting dynamic events disrupts ongoing tasks and results in prolonged waiting times.
      In the case of 55 UAVs, where HCPR exhibited longer waiting times than the CBBA, it is important to note that HCPR covered more tasks than the CBBA. The shorter waiting times reported by the CBBA were due to its inability to address tasks before they expired, resulting in fewer completed tasks overall.
  (b) **Average Number of Performed Tasks:** For small instances, as shown in Figure 12, both algorithms demonstrated an increased number of performed tasks as the number of UAVs increased, but HCPR’s growth rate and overall numbers remained superior. This suggests that HCPR’s task allocation strategies are more responsive, particularly as resources scale up. For the large dataset, shown in Figure 13, the advantage of HCPR over the CBBA became even more pronounced on the large dataset. As the map size and UAV count increased on the large dataset, HCPR maintained its lead in the number of tasks completed across both the small and large datasets. This consistency reinforces the responsiveness and adaptability of HCPR in dynamic and potentially unpredictable environments.
    The following outlines why HCPR outperformed the CBBA in terms of the number of tasks performed (as shown in Figure 12 and Figure 13):
    - Balanced Task Distribution: HCPR distributes UAVs evenly across clusters and ensures that even tasks in remote areas are completed before expiration, which leads to more tasks being performed. In contrast, the CBBA’s focus near the base station often leads to distant tasks expiring.
    - Efficient Task Reassignment: HCPR utilizes a small team of UAVs for partial reassignment processes and releases only a minor portion of their original tasks. This enables these UAVs to complete the reassignment quickly and resume their primary tasks sooner, thus executing more tasks overall. In contrast, the CBBA’s comprehensive reassignment approach involves all UAVs and their tasks, resulting in delays and fewer completed tasks.
    - Utilization of Idle UAVs: HCPR actively engages idle UAVs in handling new tasks or assisting current or nearby clusters and, as a result, the task completion rates are enhanced. The CBBA, however, restricts UAVs to their assigned task lists and neglects idle UAVs, which can lead to the expiration of many tasks.
    - Communication Efficiency: HCPR restricts communication to selected participant UAVs and the central controller, minimizes task interruptions, and allows more tasks to be completed in a timely manner before the end time. The CBBA’s extensive communication requirements for all UAVs during dynamic events cause task execution delays and higher expiration rates.
2. **Solution Scalability:** Scalability was evaluated by measuring the UAV throughput and completion time. Throughput, which accounts for time discounting, reflects the solution’s capacity to complete tasks efficiently, even as the problem scale increases, while completion time reveals the solution’s ability to finalize the mission swiftly and indicates its ability to efficiently scale up to larger or more complex operations.
  (a) **Average UAV Throughput:** For small instances, as shown in Figure 14, the throughput advantage of HCPR over the CBBA was clear across all tested scenarios. As the number of UAVs increased, the throughput for both algorithms increased. However, HCPR maintained a higher margin over the CBBA, which indicates that HCPR’s task allocation and execution strategies scale more effectively with increased resources. In the context of large instances (Figure 15), HCPR showed consistent performance improvements over the CBBA across different map sizes and UAV numbers. HCPR also not only demonstrated a higher efficiency in task execution, but also superior scalability. As the number of UAVs increased, HCPR was able to leverage the additional resources more effectively than the CBBA, leading to a significantly higher throughput in both small and large operational scenarios and suggesting that HCPR is particularly well-suited for large-scale, dynamic environments.
    Many factors explain why HCPR outperforms the CBBA in the various scenarios (Figure 14 and Figure 15):
    - Balanced Distribution and UAV Throughput: Distributing UAVs across different areas in a balanced manner positively impacts throughput. This is because tasks located further away receive timely attention before they expire, which ensures maximum reward collection. On the other hand, the CBBA concentrates UAV activity near the base station, which leads to delayed execution and reduced rewards for distant tasks, ultimately resulting in lower throughput, due to tasks expiring unattended.
    - Efficiency in Dynamic Event Handling: HCPR consistently outperformed the CBBA by selecting groups of nearby UAVs for reassignment processes, which leads to quicker negotiations and reduced travel times. This efficient management allows these UAVs to resume their tasks sooner and enhances the overall throughput. Furthermore, UAVs not involved in the event continue their tasks uninterrupted and avoid delays that negatively impact throughput. Additionally, by releasing only the farthest portion of the original assignments, the reassignment process is accomplished more quickly, further increasing the UAV throughput.
    - Utilization of Idle UAVs: Implementing an idle UAV handler that fully utilizes all available UAVs accelerates the completion of missions, increases the number of tasks performed, and consequently boosts UAV throughput.
    - Communication Restriction Benefits: Limiting communication to a few UAVs and the central controller enables most UAVs to complete their tasks with minimal interruptions and leads to greater throughput. This strategy also reduces waiting times that adversely decrease task rewards, thereby enhancing overall operational efficiency.
  (b) **Average Completion Time:** Across all map sizes and numbers of UAVs on the small datasets, shown in Figure 16, HCPR consistently achieved much lower average completion times than the CBBA. As the number of UAVs increased, the completion time for both algorithms increased, due to the complexity of coordinating more agents. However, the increase in the completion time of HCPR was much less pronounced than that of the CBBA, indicating that HCPR has superior scalability and task management efficiency. On large datasets, as shown in Figure 17, the efficiency gap between HCPR and the CBBA widened significantly. HCPR significantly enhanced the operational efficiency and enabled much faster mission completions across the various scales and complexities. This efficiency is crucial for dynamic environments, where timely task execution is essential.
    To explain why HCPR outperformed the CBBA in terms of mission completion time, as shown in Figure 16 and Figure 17, the following streamlined justifications are used:
    - Localized Management through Clustering: HCPR divides the environment into manageable clusters for localized control and faster completion times. In contrast, the CBBA treats the environment as a single unit, which is less efficient and more time consuming.
    - Efficient Task Reassignment: HCPR uses a small subgroup of UAVs for quick partial reassignments and releases only a minor portion of their tasks, which greatly speeds up the completion process. The CBBA, however, involves all UAVs and their entire task list in reassignments when new events are detected that lead to prolonged completion times.
    - Utilization of Idle UAVs: HCPR actively employs idle UAVs to assist in ongoing missions, significantly reducing completion times. The CBBA lacks a mechanism to utilize idle UAVs effectively; each UAV adheres rigidly to its assigned tasks, which can delay overall mission completion.
    - Communication Efficiency: HCPR limits communication to the necessary UAVs within clusters and to a central controller, restricts operations, and reduces mission completion times. The CBBA’s extensive communication among all UAVs leads to delays, requiring more time to complete missions.
3. **Solution Robustness:** Robustness can be measured by the number of new tasks covered, which helps estimate the solution’s robustness and adaptability to unexpected changes and additional demands, reflecting its capacity to integrate and manage new tasks effectively.
  - **Average Number of Covered New Tasks:** For small instances, as shown in Figure 18, both algorithms demonstrated the ability to cover new tasks, with HCPR generally outperforming the CBBA as the number of UAVs increased. This suggests that HCPR has a more effective mechanism for integrating new tasks, due to its dynamic reallocation strategies. For large instances, as shown in Figure 19, the robustness of HCPR became evident, with a significant increase in the number of new tasks covered across all UAV numbers and map sizes, which demonstrates the superior adaptability and robustness of HCPR to dynamic changes.
    To explain why HCPR outperformed the CBBA in covering new tasks, as shown in Figure 18 and Figure 19, concise explanations are presented as follows:
    (a) Even Distribution of UAVs: HCPR’s strategy of evenly distributing UAVs throughout the environment enhances the detection of new tasks across a broader area and prevents task expiration by ensuring timely discovery. In contrast, the CBBA focuses UAV activity near the base station, often resulting in delayed arrival and potential expiration of tasks in distant regions.
    (b) Task Reallocation Strategy: Upon detecting new tasks, HCPR instructs selected participating UAVs to release a portion of the farthest tasks. This prioritization ensures that newly detected tasks, being closer to the involved UAVs, are more likely to be allocated quickly, which enhances coverage efficiency.
    (c) Utilization of Idle UAVs: HCPR proactively checks for new task lists when UAVs become idle and immediately reallocates these UAVs to cover new tasks. Conversely, the CBBA lacks a specific strategy for utilizing idle UAVs, often leading to new tasks expiring before they can be addressed.
    (d) Communication Efficiency: HCPR’s restricted communication protocol allows UAVs to concentrate on covering new tasks with fewer interruptions, leading to more efficient task coverage compared to the CBBA’s approach, which involves extensive communication, which can disrupt task coverage.

### 5.3. Comparison of the Results with State-of-the-Art Solutions

First, the HCPR algorithm was compared with the algorithm proposed in [33]. The experiment was conducted in a search and rescue (S&R) setting, with four UAVs from the same base assigned to complete 14 tasks within a 1 square kilometer (1 km × 1 km) two-dimensional mission area. Two UAVs were assigned to search tasks with a velocity of 80 m/s and a fuel consumption rate of 8/km, and two were assigned to rescue tasks with a velocity of 60 m/s and a fuel rate of 5/km. Tasks were divided into search and rescue types, with rewards ranging from 10 to 90 and time-decay factors of 0.05 and 0.1, respectively. The task appearance interval was 5 s for search tasks and 15 s for rescue tasks. All tasks operated within a fixed time window of [0, 100] s. The score function used to measure the solution quality is given in the following Equation (7):

(7) $$\max c_{ij}\left(t_{ij},p_{i}\right)=R0_{j}+e^{-\lambda_{j}\left(t_{ij}-t_{j}^{\mathrm{start}}\right)}R_{j}u\left(t_{ij}\right)-f_{i}D_{ij}$$

Here, $t_{ij}$ represents the time taken by UAV $U_{i}$ to perform task $T_{j}$ along its designated path $p_{i}$, $\lambda_{j}$ denotes the time-discount factor for the score of task $T_{j}$, $R0_{j}$ is the fixed reward for task $T_{j}$, and $R_{j}$ is the initial reward tied to task $T_{j}$. The binary variable $u(t_{ij})$, which takes values from the set {0, 1}, indicates whether the time window (TW) constraint is satisfied for $t_{ij}$, and $D_{ij}$ is the distance between the starting location of UAV $U_{i}$ and the location of task $T_{j}$. Different dynamic events were tested in this paper:

1. **Appearance of a new task:** First, a new search task, denoted as $T^{*}$, was assumed to have emerged in the time-sensitive and dynamic search and rescue (S&R) scenario. The time window (TW) for this urgent new task $T^{*}$ was set to [20, 70] s. Table 4 shows a comparative analysis of different reassignment strategies against the HCPR algorithm in terms of their respective scores. Notably, the HCPR algorithm outperformed all listed strategies, with a score of 422.9202, which highlights its superior adaptability and optimization capabilities in dynamic environments. The superior score achieved by the HCPR algorithm compared to the other benchmarks can be attributed to several distinct features of its design:
  - While the HCPR algorithm includes a clustering mechanism for efficient area division and task management, this feature was not utilized because the dataset, which comprises four UAVs and 14 tasks, was too small, and the area was treated as a single region. Therefore, clustering did not contribute to the higher score in this instance.
  - The HCPR strategy allows only a subset of the UAVs nearest to a new task to engage in the reassignment process, which enhances efficiency. However, given that the new task was a search task and that only two UAVs in the dataset were designated searchers, both participated in the process, which made the participant selection aspect irrelevant in this scenario.
  - The HCPR algorithm optimizes task reassignment by releasing only those original tasks that are farthest from the new task, thereby minimizing the negative impact on the score function from the travel distance. In contrast to the methodology described in the paper, which selects tasks for reassignment based on overlapping time windows (TWs) with the new task, without considering their proximity, the HCPR algorithm prioritizes the release of tasks that are geographically further away from the new task, thereby ensuring that tasks with greater potential to reduce the score are prioritized for reassignment, significantly contributing to the improved score outcome.
  - An additional strength of HCPR is its management of idle UAVs; the algorithm effectively reallocates UAVs that have completed their assignments, to assist others of the same type. This proactive utilization of resources is another key element that boosted the algorithm’s score.
2. **Different TWs of a New Task** $T^{*}$: Then, we assumed that the new search task $T^{*}$ could have varying time windows (TWs), and the score increments were compared for the HCPR algorithm and the other algorithms. As shown in Table 5, the HCPR algorithm, with scores of 10.5163 for TWs [5, 25] and [5, 50], 20.2231 for TWs [10, 25] and [10, 50], and 32.5820 for TWs [15, 25] and [15, 50], significantly outperformed the strategies proposed in other papers. The HCPR showed a progressive improvement as the TW increased, since the new task was identical in location, duration, and reward, differing only in its time window (TW), thereby uniquely influencing the overall performance metric. This divergence is attributed to the algorithm’s sensitivity to task execution timings. The original tasks, which commenced at time zero, experienced delays upon the introduction of an earlier-starting new task, which detrimentally affected this performance metric. Conversely, integrating a new task with a delayed start, while preceding tasks initiated at zero, minimized the impact, preserving or potentially enhancing the performance metric. This dynamic underscores the critical role of task schedule timing in optimizing performance within the proposed framework.
3. **Continuous Appearance of** $T^{*}$: One hundred Monte Carlo simulations were conducted to analyze the appearance of five new tasks. The score improvements of HCPR and the other benchmarks are shown in Table 6. The consistent performance of the HCPR algorithm in these simulations highlights its robustness in dynamic scenarios, where multiple new tasks appear continuously. The adaptability of HCPR to efficiently integrate multiple new tasks into the ongoing mission, without significant disruptions to existing operations, contributed to its higher score outcomes. In essence, HCPR’s higher score was largely due to two key factors:
  - It employs a task release mechanism that considers the spatial positioning of tasks and favors the reassignment of tasks that are located further away. This reduces the travel distance and enhances operational efficiency.
  - The algorithm adopts the reallocation of idle UAVs that have completed their tasks, ensuring full resource utilization and enhancing the overall score value.
4. **Different Scales of S&R Scenarios:** The simulations described previously demonstrated both the feasibility and superiority of the HCPR algorithm compared to the algorithm proposed in the other papers. Subsequently, various scales of S&R scenarios were implemented to assess the scalability of the algorithms. The authors created scenarios with randomly generated original tasks, where NU is the number of UAVs and NT is the number of tasks within a 1 km × 1 km 2D mission area. The time windows (TWs) for these tasks were randomly selected within [0, 100] seconds. The score improvements from 100 Monte Carlo simulations are illustrated in Table 7. The superior performance of the HCPR algorithm compared to the other algorithms can be attributed to several optimization factors that enhance efficiency and effectiveness:
  (a) It incorporates a clustering algorithm that segments the dataset into smaller, more manageable units, which facilitates more effective task reassignment.
  (b) In contrast to the CBBA-LR algorithm, which involves all searcher UAVs in reassignment, HCPR selectively engages only a subset of UAVs—those in closest proximity to the new task. This targeted approach reduces the travel distance, thereby enhancing the overall efficiency of the score function.
  (c) HCPR strategically releases tasks that are farthest from critical zones and minimizes their impact on the algorithm’s score, unlike other algorithms that may not prioritize tasks based on task proximity.
  (d) The algorithm effectively utilizes idle UAVs by redeploying them to assist with tasks in their current or adjacent clusters. This approach optimizes the number of tasks completed, which leads to greater rewards and, consequently, a higher score.
  These factors collectively contributed to the elevated scoring metrics of HCPR and reflect a methodical and resource-efficient allocation of tasks within dynamic operational environments.

The HCPR algorithm was further compared with the Algorithm proposed in [42]. This article assumed the existence of four base stations, each equipped with two UAVs. These UAVs varied in terms of their cruising speeds and maximum operational ranges. A total of 20 tasks were considered, of which five included time window constraints. The additional simulation parameters are detailed in the paper. The evaluation matrix used to measure the solution quality is given by the following Equation (8):

(8) minfc=α·CT+β·AT

Here, CT represents the mission completion time, which is the moment when the UAV completed its final task. AT denotes the average flight time, defined as the average duration each UAV spent from departure to return to the platform (excluding any UAVs that sustained damage). Additionally, α and β are proportional coefficients within the range [0, 1] that add up to 1, with both coefficients set to 0.5 in this scenario. Different dynamic events were tested as follows:

1. **UAV Failure:** The UAVs depart from the base stations at t=0. At t=1200, UAV U1 was compromised by an enemy attack and can no longer perform its tasks. The TS-DTA score (fc) after UAV failure was 2051.28, whereas the HCPR score was 1018.
2. **The Appearance of a New Task:** At t=1300, the early warning UAV detected three new tasks. The TS-DTA score (fc) after UAV failure was 2166.98, while the corresponding HCPR score was 1288.7.

In scenarios (1) and (2), HCPR did not utilize the partial reassignment process, and task clustering had no effect. The reason for the superiority of HCPR compared with TS-DTA was the deployment of an idle UAV handler, since one of the UAVs was not assigned in the initial assignment and thus was available to handle the detected new tasks and all unperformed tasks for the failed UAV.

**Figure 20:**
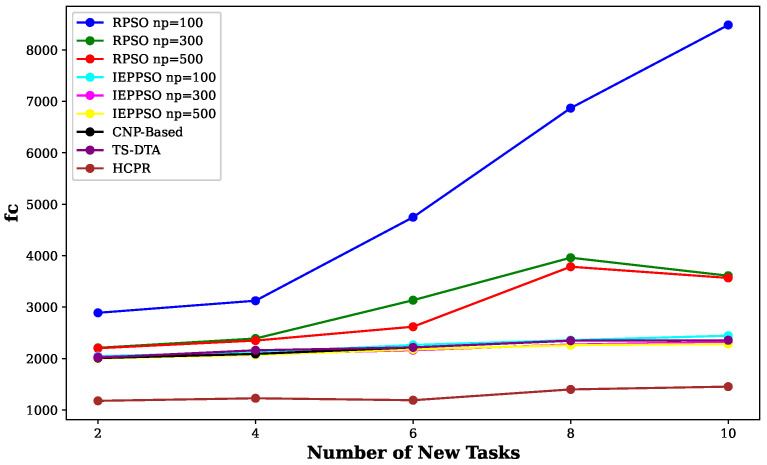
Performance comparison between HCPR and other algorithms presented in [42].

**Comparative Analysis of Solution Quality:** We conducted a comparative analysis of the HCPR algorithm against TS-DTA [42], CNP-based [43], and two centralized task assignment algorithms, RPSO [48] and IEPPSO [49]. These comparisons were initially based on the occurrence of a single dynamic event—the appearance of a new target. For a more comprehensive analysis, we introduced various numbers of new targets into Experiments 2, 4, 6, 8, and 10. We assessed the performance by evaluating the overall cost of UAV formation (fc) across different scenarios and depicted these variations in a line chart (Figure 20) that illustrates how the overall cost changed with the number of targets allocated. HCPR significantly outperformed the other algorithms in minimizing the fc function across the various scenarios, consistently achieving the lowest fc values.

## 6. Conclusions

This study introduced a novel hybrid algorithm to address the multi-UAV dynamic task assignment problem in dynamic and large-scale environments. The proposed algorithm integrates clustering-based task allocation with a partial reassignment strategy, enabling efficient task distribution, robust adaptability to dynamic events, and enhanced scalability. Through extensive simulations, the algorithm demonstrated superior performance over existing solutions, particularly in reducing task waiting times, increasing UAV throughput, improving task completion rates, and efficiently handling new dynamic events. These results validate the algorithm’s effectiveness in improving responsiveness, robustness, and scalability in dynamic UAV operations. The significance of this research lies in its ability to bridge critical gaps in multi-UAV task assignment by optimizing UAV coordination, while minimizing communication overhead. The proposed approach holds promise for applications in disaster response, surveillance, search and rescue, and large-scale infrastructure inspections, where real-time task allocation and adaptability are essential.

Future research can extend this work by incorporating machine-learning-based predictive models to anticipate dynamic changes, integrating heterogeneous UAVs with varying capabilities, and exploring real-world implementations to validate the model beyond simulations. Further enhancements could also focus on optimizing the balance between centralized and distributed decision-making, to improve resilience in communication-constrained environments. This study provides a solid foundation for advancing multi-UAV task allocation strategies, offering a scalable and efficient solution for dynamic and mission-critical UAV operations. 

## Figures and Tables

**Table 1:** Symbol definitions.

Symbol	Definition
*T*	Set of tasks $\left\{T_{1},T_{2},T_{3},\ldots ,T_{M}\right\}$
nT	Set of unknown new tasks
*U*	Set of UAVs $\left\{U_{1},U_{2},U_{3},\ldots ,U_{N}\right\}$
$a_{i}$	The assigned tasks list of UAV $U_{i}$, where $a_{i}=\left[T_{1},T_{2},\ldots ,T_{|a_{i}|}\right]$
*M*	Number of tasks
*N*	Number of UAVs
*Q*	Number of new tasks
nC	Number of Clusters
*i*	The UAVs index with the range 1≤i≤N
*j*	The tasks index with the range $1\leq j\leq |a_{i}|$
*q*	The new tasks index with the range 1≤j≤Q
$s_{i,j}$	The score value of UAV $U_{i}$ performing task $T_{j}$
$ts_{j}$	The start time of task $T_{j}$
$te_{j}$	The end time of task $T_{j}$
$\tau_{i,j}$	The execution time of task $T_{j}$ by $U_{i}$
$L_{i}$	The maximum number of tasks that can be performed by $U_{i}$
$R_{j}$	The task reward of $T_{j}$
$d_{0,j}$	The distance from the base station to task $T_{j}$
$d_{i,j}$	The distance from task $T_{i}$ to task $T_{j}$
$v_{i}$	The velocity of $U_{i}$
$D_{j}$	The duration of task $T_{j}$
λ	Time discount factor
$c_{i,j}$	The waiting time of task $T_{j}$ until executed by $U_{i}$
$th_{i,j}$	The gained throughput of UAV $U_{i}$ after performing task $T_{j}$
np	Number of participated UAVs in reassignment process
nr	Number of released tasks during reassignment process
mpS	Width of the simulation map
dist	Task distribution, which can be random, concentrated, or predetermined

**Table 2:** Experimental setup.

Instances Size	Map Size	#Tasks (*M*)	#UAVs (*N*)
Small	1000-1250-1500-1750-2000	100-150-200-250-300	5-7-10-12-15
Large	6000-7000-8000-9000-10,000	600-700-800-900-1000	35-40-45-50-55

**Table 3:** Predefined parameters.

Parameter	Value (Small and Large Instances)
#Clusters	Small: 2, 3, 4, 5, 6 | Large: 12, 13, 14, 15, 16
UAVs Max Load	M/N
UAVs Velocity	20% MapSize
#New Tasks	5% *M*
Sensor Detection Range	7% MapSize
Task Time Window	[rand×5%M,ts+D+rand×60%M]

**Table 4:** Task scores recorded for various algorithms after the appearance of a new task. Bold text indicates the highest score.

Algorithms	Score
No reset [31]	380.7479
Full reset [34]	382.0065
Single reset [35]	381.3196
PR with fixed reset [35]	382.0065
PR with team reset [35]	382.0065
CBBA-LR [33]	382.0065
HCPR	**422.9202**

**Table 5:** Absolute task scores for different time windows (TW) for a newly introduced task. Bold text indicates the highest score.

Algorithms	Time Window (TW)					
	**[5, 25]**	**[5, 50]**	**[10, 25]**	**[10, 50]**	**[15, 25]**	**[15, 50]**
No reset [31]	0	5.7042	0	5.7042	0	5.7042
Full reset [34]	6.9627	6.9627	6.9627	6.9627	6.9627	6.9627
Single reset [35]	0	5.7042	0	5.7042	0	5.7042
Heuristic full reset [35]	6.9627	6.9627	6.9627	6.9627	6.9627	6.9627
PR with fixed reset [35]	6.9627	6.9627	6.9627	6.9627	6.9627	6.9627
PR with team reset [35]	6.9627	6.9627	6.9627	6.9627	6.9627	6.9627
CBBA-LR [33]	6.9627	6.9627	6.9627	6.9627	6.9627	6.9627
HCPR	**10.5163**	**10.5163**	**20.2231**	**20.2231**	**32.5820**	**32.5820**

**Table 6:** Absolute task score result of continuous appearance of new tasks (T*). Bold text indicates the highest score.

Algorithms	One New Task	Two New Tasks	Three New Tasks	Four New Tasks	Five New Tasks
No reset [31]	5.1589	6.8019	10.2410	12.5276	14.384
Full reset [34]	8.5182	10.2016	16.6892	18.846	22.7084
Single reset [35]	5.2262	6.8019	10.2410	12.5276	14.384
Heuristic full reset [35]	8.5182	10.2016	15.2255	17.5712	19.9235
PR with fixed reset [35]	8.5182	10.2016	15.2255	17.820	20.1234
PR with team reset [35]	8.5182	10.2016	15.2255	17.820	20.1234
CBBA-LR [33]	8.5182	10.2016	16.6892	18.846	22.7084
HCPR	**21.575518**	**41.780054**	**72.875293**	**87.511316**	**103.221674**

**Table 7:** Task score values obtained by HCPR and the other algorithms across different scales of S&R scenarios. Bold text indicates the highest score.

Scenario	No [31]	Full [34]	Single [35]	Heuristic [35]	Fixed [35]	Team [35]	CBBA-LR [33]	HCPR
NU = 5, NT = 10	6.8650	9.1492	6.8650	8.6004	8.3589	8.3589	9.1492	**12.456548**
NU = 5, NT = 20	3.8654	5.1698	3.8654	5.1698	4.3984	4.3984	5.1698	**10.703387**
NU = 5, NT = 30	2.0211	4.8586	2.0211	4.7583	2.8516	2.6798	4.8586	**11.454415**
NU = 10, NT = 20	9.3835	10.9559	9.3835	10.4736	10.7176	10.3445	10.9559	**18.517290**
NU = 10, NT = 30	6.4906	8.9158	6.4906	8.3174	8.1911	8.2538	8.9158	**11.712061**
NU = 10, NT = 40	4.6215	7.7695	4.6215	7.5584	5.9541	5.0388	7.7695	**10.413622**
NU = 15, NT = 30	9.0957	11.3661	9.0957	10.2369	11.0944	10.8700	11.3661	**17.486393**
NU = 15, NT = 40	7.5692	10.0984	7.5692	9.2497	9.1497	8.6179	10.0984	**17.123623**
NU = 15, NT = 50	6.2113	7.4322	6.2113	7.3528	7.1030	6.3295	7.4322	**11.635331**
NU = 15, NT = 60	4.9995	6.8825	4.9995	6.8344	5.6678	4.8690	6.8825	**9.987903**
NU = 20, NT = 40	9.3634	12.3817	9.3634	10.5540	12.1520	10.7517	12.3817	**23.528094**
NU = 20, NT = 50	8.3122	10.4728	8.3122	9.6811	10.1067	8.7328	10.4728	**19.281718**
NU = 20, NT = 60	7.2814	11.1660	7.2814	9.7506	10.0227	7.2916	11.1660	**18.284739**
NU = 20, NT = 80	5.4008	8.0004	5.4008	7.7693	6.5903	5.4262	8.0004	**16.604281**
NU = 20, NT = 100	4.6560	6.1548	4.6560	6.0738	4.9219	4.6684	6.1548	**11.543217**
NU = 25, NT = 50	9.9358	12.9114	9.9358	12.8417	12.8963	12.6168	12.9114	**28.909494**
NU = 25, NT = 60	9.1387	11.7833	9.1387	10.2684	11.6273	11.6594	11.7833	**23.188391**
NU = 25, NT = 80	8.3721	9.7330	8.3721	8.3692	9.2030	8.6084	9.7330	**18.165586**
NU = 25, NT = 100	6.2311	8.5242	6.2311	8.1836	7.4738	6.9631	8.5242	**15.904294**

## Data Availability

All data used in this study are included in the article, which details the simulation parameters and configurations for result replication.
